# New Targeted Gold Nanorods for the Treatment of Glioblastoma by Photodynamic Therapy

**DOI:** 10.3390/jcm8122205

**Published:** 2019-12-13

**Authors:** Zahraa Youssef, Nurlykyz Yesmurzayeva, Ludivine Larue, Valérie Jouan-Hureaux, Ludovic Colombeau, Philippe Arnoux, Samir Acherar, Régis Vanderesse, Céline Frochot

**Affiliations:** 1 Laboratoire Réactions et Génie des Procédés (LRGP), UMR 7274, CNRS, Université de Lorraine, 54000 Nancy, France; zahraa.youssef@univ-lorraine.fr (Z.Y.); nurlykyz@mail.ru (N.Y.); ludivine.larue@univ-lorraine.fr (L.L.); lcolombeau@gmail.com (L.C.); philippe.arnoux@univ-lorraine.fr (P.A.); 2 Kazakh National Research Technical University after K.I Satpayev, 22 Satpayev str., Almaty 050013, Kazakhstan; 3 Université de Lorraine, CNRS, CRAN UMR 7039, F-54000 Nancy, France; valerie.jouan-hureaux@univ-lorraine.fr; 4 Laboratoire de Chimie Physique Macromoléculaire (LCPM), UMR 7375, CNRS, Université de Lorraine, 54000 Nancy, France; samir.acherar@univ-lorraine.fr (S.A.); regis.vanderesse@univ-lorraine.fr (R.V.)

**Keywords:** gold nanorods, photosensitizer, peptide, PDT, glioblastoma, NRP-1, targeting

## Abstract

This study describes the employment of gold nanorods (AuNRs), known for their good reputation in hyperthermia-based cancer therapy, in a hybrid combination of photosensitizers (PS) and peptides (PP). We report here, the design and the synthesis of this nanosystem and its application as a vehicle for the selective drug delivery and the efficient photodynamic therapy (PDT). AuNRs were functionalized by polyethylene glycol, phototoxic pyropheophorbide-a (Pyro) PS, and a “KDKPPR” peptide moiety to target neuropilin-1 receptor (NRP-1). The physicochemical characteristics of AuNRs, the synthesized peptide and the intermediate PP-PS conjugates were investigated. The photophysical properties of the hybrid AuNRs revealed that upon conjugation, the AuNRs acquired the characteristic properties of Pyro concerning the extension of the absorption profile and the capability to fluoresce (Φ_f_ = 0.3) and emit singlet oxygen (Φ_Δ_ = 0.4) when excited at 412 nm. Even after being conjugated onto the surface of the AuNRs, the molecular affinity of “KDKPPR” for NRP-1 was preserved. Under irradiation at 652 nm, in vitro assays were conducted on glioblastoma U87 cells incubated with different PS concentrations of free Pyro, intermediate PP-PS conjugate and hybrid AuNRs. The AuNRs showed no cytotoxicity in the absence of light even at high PS concentrations. However, they efficiently decreased the cell viability by 67% under light exposure. This nanosystem possesses good efficiency in PDT and an expected potential effect in a combined photodynamic/photothermal therapy guided by NIR fluorescence imaging of the tumors due to the presence of both the hyperthermic agent, AuNRs, and the fluorescent active phototoxic PS.

## 1. Introduction

Globally, according to the World Health Organization (WHO), cancer is listed to be the second foremost source of mortality, harvesting the life of one in six humans worldwide in 2018 [1]. This fact triggered the efforts of the scientific world in their combat against this malignant disease. Among other conventional therapies such as surgical resection, chemotherapy, radiotherapy, etc., photodynamic therapy (PDT) started to gain an increased interest over the past years as a promising clinical treatment [2,3]. PDT is a well-established minimally invasive technique for the treatment of various maladies including cancer. The three main building blocks of PDT are the photoactivatable photosensitizer (PS), light and molecular oxygen [4,5,6,7]. In the presence of an illumination source at a suitable wavelength, the PS gets excited and transfers its energy to the neighboring oxygen, that in its turn gets excited from the inactive triplet state to create singlet oxygen (^1^O_2_) or others types of reactive oxygen species (ROS). The generated ROS lie behind the cancer cell killing which is achieved by necrosis or apoptosis [5,8,9,10,11]. The key to an efficient PDT is the prolonged circulation of the drug in the blood and their specific accumulation inside the tumor [12,13]. Most PSs, described so far in the literature, belong to the hematoporphyrin family. They can produce ^1^O_2_ at high quantum yields and exhibit fluorescence that enables photodynamic imaging (PDI). However, these lack selectivity and present high hydrophobicity that limits their solubility in biological media. This issue leads to the aggregation of the PSs that, consequently, suppresses their photophysical properties especially the production of ^1^O_2_ [4,5,14,15]. To overcome this burden, and taking into account that it is hard to create a free PS with high selectivity, various nanoparticle (NP)-based carrier systems were developed [16,17,18,19,20]. These can facilitate the transportation of the dye and deliver the latter specifically to the targeted neoplastic cells due to their small size and especially their favored buildup in tumors thanks to the enhanced permeation and retention effect (EPR) [21]. The properties and composition of the NPs are tunable in a way that widens the range of their application to achieve the targeting of the tumors. NPs can be organic (liposomes, micelles, microspheres), inorganic silica-based, semi-conductors (TiO_2_, ZnO), magnetic (Fe_2_O_3_), metallic (silver, gold in its nanoparticle and nanorod shapes), cyclodextrins etc. [4,5,18,19,20,21,22,23]. In addition, some NPs have been designed to combine PDT and photothermal therapy (PTT), thus improving cancer treatment [24,25,26,27,28,29].

Gold-nanorods (AuNRs), are rod-shaped nanosized anisotropic materials that have grasped wide levels of attention due to their unique optical and photophysical properties [30,31]. These properties are granted to the AuNRs due to their intense surface plasmon bands; transverse and longitudinal bands in the visible and the near-infrared (NIR) ranges. On this basis, the AuNRs are capable of absorbing, fluorescing, and scattering light in the NIR region, thus provoking two-photon luminescence [32]. In addition, AuNRs possess a high X-ray absorption capacity, and serve as a radiosensitizing agent and a contrast agent in computed tomography scan [31]. They have been employed in various biological applications including gene delivery, cell imaging, and photothermal therapy (PTT). These AuNRs are also familiar as drug delivery vehicles in cancer diagnostics and therapy [33,34,35,36,37,38,39,40,41,42,43]. The surface of the gold nanoparticles, in general, can be easily functionalized, thus establishing very stable conjugations [44]. This fact encouraged the linking of different PSs onto the surface of the AuNPs or NRs for a PDT or a combined PDT/PTT treatment, where they showed good efficiency [45,46,47,48]. Gold NPs can be designed in different architectures (nanochains, nanorods, nanospherical, nanocages, nanotubes, etc.,) and in different sizes. Any change in one of these parameters can alter the destined application and the efficiency of these NPs in fulfilling their function [46]. However, AuNPs and NRs present certain cytotoxicity that arises from the use of cetyltrimethylammonium bromide (CTAB) and silver ions during their synthesis. The resultant cytotoxicity hinders their use in the bioscience domain [31,32]. To overcome these hindrances, the customization of the ligands onto the AuNRs, such as their conjugation with polyethylene glycol (PEG), can effectively lower the cytotoxicity [49,50]. In PDT, loading PSs onto AuNRs or AuNPs mediate a better solubilization of the former in polar media without losing its phototoxic effect. In such conjugated systems, passive targeting by EPR is established due to the nano-component, while maintaining the good efficiency of the PS in PDT [14]. The efficacy could be further boosted by combining an additional agent that guarantees an active cancer-cell-targeting [44]. The peptides can target certain over-expressed receptors onto the tumor cells themselves or the neovessels feeding the tumor via angiogenesis. Neuropilin-1 (NRP-1) is a co-receptor of vascular endothelial growth factor receptors (VEGFR) localized not only on angiogenic endothelial cells but also onto tumoral cells such as glioblastoma cell lines (U87) [51]. In attempt to destroy glioblastoma, which is the most frequent and malignant brain tumor, several peptides were developed by our team [52,53,54] and other groups [55,56,57] that successfully targeted NRP-1. There are many publications dealing with PDT and gold nanoparticles. Nevertheless, all are different, as described recently by the teams of D. Russell [58] and C.M. Dong [59]. In the literature, only four publications dealing with gold and NRP-1 targeting can be found [60,61,62,63] but, as far as we know, no publication describes the targeting of NRP-1 with gold NP in the field of PDT.

To our knowledge, very few research efforts employed the pyropheophorbide-a PS with gold nanosystems [64,65,66,67] and none targeted NRP-1with KDKPPR peptide. Since both Pyro and KDKPPR are considered interesting in PDT and targeting, respectively, in addition to previously mentioned advantages of the use of AuNRs, we went on with this challenge. This study highlights the design, including the synthesis and the characterization, of a novel hybrid AuNR-system and its application in targeted-PDT. The AuNRs were synthesized by seed-mediated method and the CTAB were substituted by HS-PEG2K-NH_2_ via ligand exchange to render biocompatible materials. A PP-PS conjugate was synthesized by the coupling of H-DKPPR-resin and pyropheophorbide-a PS together through a lysine (K) amino acid. A spacer, 6-maleimidohexanoic acid (MI), was coupled to the PP-PS conjugate that was finally grafted onto the surface of the AuNRs via click chemistry. The hybrid AuNRs were characterized physicochemically and photophysically and then tested for their affinity towards NRP-1 estimated in terms of IC_50_. The cyto- and phototoxicity of the AuNRs were also assessed in vitro on glioblastoma U87 cells.

## 2. Experimental Section

### 2.1. Chemicals and Materials

Milli-Q water was used in all the experiments. Dichloromethane (DCM), dimethylformamide (DMF), chloroform (CHCl_3_) and ethanol (EtOH) were obtained from Sigma-Aldrich (Saint-Quentin Fallavier, France) and were used without further purification.

- Fmoc-K(Pyro)-OH

For the synthesis of Fmoc-K(Pyro)-OH, pyropheophorbide-a (Pyro) photosensitizer (PS) was purchased from BOC Sciences (Shirley, NY, USA). *N*-hydroxysuccinimide (HOSu), *N*-(3-dimethylaminopropyl)-N’-ethylcarbodiimide hydrochloride (EDCI), trimethylamine (Et_3_N) and Fmoc-Lys-OH hydrochloride were obtained from Sigma Aldrich, Saint-Quentin Fallavier, France.

- Peptide synthesis

The synthesis of DKPPR-OH peptide and the corresponding Fmoc-K(Pyro)DKPPR-OH and MI-K(Pyro)DKPPR-OH platforms required 2-(1H-Benzotriazol-1-yl)-1,1,3,3-tetramethyluronium hexafluorophosphate (HBTU, Iris Biotech GmbH, Marktredwitz, Germany), *N*-methylpyrrolidine (NMP, PROLABO, Rhone-Poulenc, Paris, France), *N*-methylmorpholine (NMM, Alfa-Aesar GmbH, Karlsruhe, Germany), acetic anhydride, piperidine (Sigma-Aldrich, Saint-Quentin Fallavier, France), as reagents. Fmoc-*L*-Aspartic acid(tBu)-OH (Senn Chemicals AG, Dielsdorf, Switzerland), Fmoc-*L*-Lysine(Boc)-OH (Iris Biotech GmbH, Germany), Fmoc-*L*-Proline-OH and Fmoc-*L*-Arginine(Pbf)-Wang resin (100–200 mesh) were obtained from Merck-Novabiochem, Darmstadt, Germany. Trifluoroacetic acid (TFA), triisopropylsilane (TIPS), and 6-maleimidohexanoic acid (MI) was purchased from Sigma Aldrich (Saint-Quentin Fallavier, France).

- Gold nanorods

For the synthesis of the gold nanorods, cetyltrimethylammonium bromide (CTAB, 98%), sodium tetrachloroaurate(III) dehydrate (NaAuCl_4_·2H_2_O), sodium borohydride (NaBH_4_, 99%), silver nitrate (AgNO_3_), *L*-ascorbic acid, HS-PEG2K-NH_2_, Tween 20 (Polysorbate 20), sodium chloride (NaCl) and 2-iminothiolane.HCl (Traut’s reagent) were purchased from Alfa Aesar, Karlsruhe, Germany.

- In vitro studies

Hanks’ Balanced Salt solution (HBSS buffer), trypsin, 3-(4,5-Dimethylthiazol-2-yl)-2,5-diphenyl tetrazolium bromide (MTT), Fetal Calf Serum (FCS), antibiotic (penicillin-streptomycin), fluorescamine, and ninhydrin were purchased from Sigma-Aldrich, Saint-Quentin Fallavier, France. Dulbecco’s Modified Eagle’s Medium (DMEM) was obtained from Gibco BRL, Illkirch-Graffenstaden, France. U87 MG glioblastoma cell lines (HTB-14TM) were provided by ATCC, LGC Molsheim, France. NRP-1, VEGF-A165, streptavidin horseradish peroxidase conjugate, tetramethylbenzidine and H_2_O_2_, Subtrate reagent pack were from Bio-Techne, Noyal-Châtillon-sur-Seiche, France.

### 2.2. Preparation of the Gold Nanorods (AuNRs)

#### 2.2.1. Synthesis of the CTAB-Capped AuNRs (AuNRs@CTAB)

Gold nanorods were synthesized, with minor modifications, by the seed-mediated method described by El-Sayed’s group [68]. In a typical protocol, all the glassware was washed with aqua regia (3HCl:HNO_3_) and rinsed with the proper amount of deionized water. To formulate the seed solution, a mixture of NaAuCl_4_·2H_2_O (5 mL, 0.5 mM) and CTAB (5 mL, 0.2 M) was prepared. To this mixture, ice-cold NaBH_4_ solution (0.6 mL, 0.01 M) was added and a brownish yellow solution was formed. The solution was intensely stirred for 2 min, then incubated for 2 h at 30 °C. The growth solution was produced by the successive addition of AgNO_3_ (1.5 mL, 4 mM), NaAuCl_4_·2H_2_O (30 mL, 1 mM) and ascorbic acid (0.42 mL, 78.8 mM) into a solution of CTAB (30 mL, 0.2 M). The presence of ascorbic acid, which is a mild reducing agent, caused the immediate change in the color of the solution from dark yellow to colorless. In total, 72 µL of the seed solution was added into the growth solution and the obtained mixture was incubated overnight at 30 °C. The color of this mixture turned to vinous. The gold nanorods were washed thoroughly and recovered by centrifugation (13,093× *g*, 3 cycles) and then re-dispersed in milli-Q water.

#### 2.2.2. PEGylation of AuNRs (AuNRs@PEG)

HS-PEG2K-NH_2_ was used as the pegylation agent of the gold nanorods. The synthesis protocol included the addition of Tween 20 (5 µL, 2 vol%) as a stabilizer agent, NaCl (50 µL, 2 M) to etch the silver, deionized water (30 µL) and HS-PEG2K-NH_2_ (12.6 µL, 1.6 mM, Mw≈2000) into a 100 µL solution of concentrated AuNRs (optical density OD of 9). The mixture was then agitated for 24 h at room temperature in a shaker (frequency 550 rpm). Afterwards, the AuNRs@PEG were centrifuged, then washed and recovered by centrifugation at 13,093× *g* to eliminate the unreacted HS-PEG2K-NH_2_. The obtained AuNRs were redispersed in milli-Q water. The quantification of PEG chains per AuNRs was performed using both fluorescamine- and ninhydrin-based assays where the resultant supernatant was exposed to these assays to determine the number of unreacted HS-PEG2K-NH_2_ molecules [69].

- Fluorescamine-Based Assays

Firstly, different solutions of HS-PEG2K-NH_2_ were prepared at known concentrations in phosphate buffered solution (PBS) at a basic pH (pH = 10). A solution of 2 µM of fluorescamine was also prepared in acetone. To 3 mL of each of the PEG solutions, 0.25 mL of the fluorescamine solution were added and kept for 15 min. The fluorescence intensity of each solution was measured at 480 nm upon excitation at 390 nm. On this basis, a calibration curve of the fluorescence intensities as a function of the concentration of the solutions was plotted. The fluorescence intensity of the supernatant was measured by diluting 100 µL of this solution in 3 mL of PBS and treated similarly to the solutions used for the plotting of the calibration curve. The corresponding concentration of the HS-PEG2K-NH_2_ was calculated from the calibration curve taking into account the dilution factor. The quantity of PEG that occupied the surface of the AuNR was calculated by subtracting that initial concentration of the solution to that obtained from the calibration curve and estimated in terms of number of PEG molecules per AuNR.

- Ninhydrin-Based Assays

The preparation of all the reagents that we have used for this assay is described in the literature [69]. Firstly, different solutions of HS-PEG2K-NH_2_ were prepared at known concentrations in PBS. To 250 µL of each of the PEG solutions, 100 μL of 6% ninhydrin/ethanol solution and 200 μL of KCN/phenol solution were added and were heated at 100 °C for 4 min. After cooling, 200 μL of 60 wt% ethanol in water was added. The UV-vis absorption of each solution was measured and a calibration curve of the absorption at 565 nm as a function of the concentration of the solutions was plotted. The absorption of the supernatant was measured by treating 250 µL with the same procedure used for the standard solutions. Similarly, to fluorescamine-based assay, the corresponding concentration of the HS-PEG2K-NH_2_ was calculated from the calibration curve taking into account the dilution factor. The quantity of PEG that occupied the surface of the AuNRs was calculated by subtracting that initial concentration of the solution to that obtained from the calibration curve and estimated in terms of number of PEG molecules per AuNR.

#### 2.2.3. Thiolation of the AuNRs@PEG

Thiolation of the AuNRs@PEG by Traut’s reagent was performed to effectuate the final coupling of peptide-photosensitizer conjugate (noted PP-PS) to the AuNRs. Traut’s reagent reacts with primary amines to introduce sulfhydryl groups. A solution of AuNRs@PEG with an OD adjusted at 9 was prepared in water at a pH of 7.64 and kept at room temperature for 1 h. 0.02 mmol of Traut’s reagent was dissolved in water and added into the solution of the AuNRs@PEG to afford AuNRs@PEG-SH. After 1 h, the unreacted Traut’s reagents were removed from the solution of the thiolated NRs through several washings and centrifugation (13,093× *g*, 4 times). The degree of the purity was controlled by recording the absorbance spectra of the supernatant at 248 nm, which corresponds to the Traut’s reagent.

### 2.3. Synthesis of the MI-K(Pyro)DKPPR

#### 2.3.1. Synthesis of Pyro-OSu

In the dark and under inert conditions, an equivalent of Pyro was dissolved in THF/DMF (80/20, v/v). There were 1.5 equivalents of each of HOSu and EDCI added. The reaction mixture was kept under stirring at 45 °C overnight. The evolution and the termination of the reaction was traced by thin layer chromatography (TLC) method. The crude product was further purified by silica gel column with DCM/EtOH (90/10, v/v) as the eluent. The solvents were evaporated and the Pyro-OSu was obtained as a green solid product.

#### 2.3.2. Synthesis of Fmoc-K(Pyro)-OH

In the dark and under inert conditions, an equivalent of Pyro-OSu (0.2 mmol, 126 mg) was dissolved in 10 mL CHCl_3_. Then, 1.2 equivalents of Fmoc-Lys-OH (0.24 mmol, 97 mg) were dissolved in 1 mL of DMF in the presence of 2.4 equivalents of triethylamine (0.48 mmol, 61 µL). These components were then added to the Pyro-OSu solution. The reaction mixture was stirred for 24 h at room temperature. The evolution of the reaction was monitored by TLC. The solvents were evaporated and the crude product was purified by silica gel chromatography using DCM/EtOH (95/5, v/v) as the eluent. The pure product was collected as a green solid powder.

#### 2.3.3. Synthesis of H-DKPPR-Wang Peptide

As previously described [70], the H-DKPPR-Wang was constructed on a Fmoc-Arg(Pbf)-Wang resin as starting material using an automated ResPep XL peptide synthesizer (Intavis AG, Bioanalytical Instruments, Köln, Germany) and operated with a Multiple-Parallel Peptide Synthesis Program at a 100 μmol synthesis scale. The iterative couplings of amino acids were performed through a classical SPPS Fmoc/tBu protocol with 3 equiv of *N*-Fmoc-amino acid, 3 equiv of HBTU as activating reagents, and 9 equiv of NMM in a minimum of DMF (2 mL). The side chains of aspartic acid (Asp, D), lysine (Lys, K) and arginine (Arg, R) were respectively protected by 2,2,4,6,7-pentamethyldihydrobenzofuran-5-sulfonyl (Pbf), tert-butyl ester (OtBu) and *N*-tert-butyloxycarbonyl (Boc), respectively. Fmoc deprotection was realized using a 20% piperidine solution in DMF. The unreacted amino acids were capped with acetic anhydride at the end of each coupling phase.

#### 2.3.4. Synthesis of Fmoc-K(Pyro)DKPPR-Wang Peptide

Fmoc-K(Pyro)DKPPR-resin was synthesized on solid phase, between the *N*-terminal of the H-DKPPR-resin and the carboxyl group of the Fmoc-K(pyro)-OH building block. To an equivalent of H-DKPPR-resin dissolved in DMF, 1.5 equivalents of Fmoc-K(Pyro)-OH, 3 equivalents of HBTU, 9 equivalents of NMM, and 3 equivalents of NMP were added. The reaction was conducted for 24 h at room temperature under gentle stirring. The resin was washed with DCM and DMF to remove unreacted reagents and Fmoc-K(Pyro)DKPPR-resin was successfully obtained and kept conserved on the resin for subsequent reaction on solid phase.

#### 2.3.5. Synthesis of MI-K(Pyro)DKPPR-OH

The Fmoc deprotection of Fmoc-K(Pyro)DKPPR-resin conjugate was realized using a 25% piperidine solution in DMF. 6-Maleimidohexanoic acid (MI) was coupled to the deprotected *N*-terminal of the peptide-resin. Briefly, to an equivalent of H-DKPPR-resin dissolved in DMF, 1.2 equivalents of MI, 3 equivalents of HBTU, 9 equivalents of NMM and 3 equivalents of NMP were added. The reaction was conducted for 24 h at room temperature. The resin was then washed with DMF, ethanol, and DCM before cleavage. Trifluoroacetic acid (TFA) cleavage was conducted on the synthesized MI-K(Pyro)DKPPR-Wang peptide following a standard protocol with triisopropylsilane (TIPS) as the scavenger (95% TFA: 2.5% TIPS: 5% Water) while shaken for 2 h at room temperature. The cleaved MI-K(Pyro)DKPPR-OH peptide was then precipitated in cold diethyl ether, washed several times and recovered by centrifugation (4000 rpm, 4 cycles). The crude product was further purified by preparative HPLC using acetonitrile/water (0.1% TFA; 10:90) to 100% acetonitrile gradient in 15 min, followed by isocratic acetonitrile for 10 min at a flow of 4 mL/min (Rt = 17.8 min). Pure product was isolated as a green powder (50 mg, 21%).

### 2.4. Coupling of the MI-K(Pyro)DKPPR-OH Conjugate to the AuNRs@PEG-SH

The coupling of the MI-K(Pyro)-DKPPR-OH conjugate to the surface of the AuNRs@PEG-SH was performed via the maleimido-thiol click chemistry. The AuNRs@PEG-SH were dispersed in PBS (0.1 M, pH = 8) at room temperature. The MI-K(Pyro)DKPPR-OH was dissolved in DMSO (3 mg/mL) and then added to the AuNRs@PEG-SH dispersion and was kept under stirring for 8 h in the dark. The purification of the mixture was carried out by ultracentrifugation (13,093× *g*, 3 times). The resulting AuNRs@PEG-MI-K(Pyro)DKPPR-OH were eventually freeze-dried for further storage.

### 2.5. Physico-Chemical and Photophysical Characterizations

The transmission electronic microscopy (TEM) imaging was realized on a JEOL Atomic Resolution 200F cold FEG (TEM / STEM) instrument. The samples were prepared by administering the suspension of AuNRs onto a 300-mesh Formvar-coated copper grid that had been previously hydrophilized under UV light (Electron Microscopy Sciences, Hatfield, PA, USA).

The evolution in the charge of the AuNRs in the absence or presence of PEG chains (zeta-potential) was determined using a Zetasizer NanoZS equipped with a He-Ne laser at 633 nm (Malvern). The AuNRs were first dispersed in milli-Q water to be then diluted in an aqueous solution of NaCl (10^−2^ M). Raman spectra were collected on a HORIBA XploRA spectrometer and an OLYMPUS BX51 microscope. For all Raman measurements, the samples were exposed to a near-infrared diode laser (λ_ex_ = 785 nm, P_ex_ = 44 mW) for 3 s, and the Raman signals were collected from 5 scans at 1800 T (1800 lines/mm). Elemental analyses were conducted using X-ray photoelectron spectroscopy (XPS). A Kratos Axis ULTRA XPS incorporating 165 mm hemispherical electron energy was used. The incident radiation was monochromatic AlKalpla Xrays (1486.6 eV) at 225 W (15 kV, 8 ma). Survey (wide) scans were taken at an analyzer pass energy of 160 eV and multiplex (narrow) higher resolution scans at 20 eV. Survey scans were carried out over 1100 eV binding energy with 1.0 eV steps and a dwell time of 100 ms. Binding energy were calibrated with C 1s peak at 284.6 eV to compensate for surface charging effect. Component fitting of the high-resolution spectra was performed using Kratos X-Vision 2.2.11 software (San Diego, CA, USA). ^1^H NMR spectra were recorded on a Brucker Advance 300 MHz spectrophotometer (Saarbrücken, Germany). The spectra were recorded in CDCl_3_ or DMSO-d_6_ at room temperature (T = 298 K) using TMS (δ = 0 ppm) or residual DMSO (δ = 2.5 ppm) as internal references. The chemical shifts (δ) are given in parts per million (ppm). The multiplicities and abbreviations are: s = singlet, d = doublet, t = triplet, q = quadruplet, m = multiplet, br = large and Ar = aromatic. The mass spectra were recorded on a Schimadzu LC-MS 2020 mass spectrometer by positive electrospray ionization. High resolution mass spectrometry (HRMS) experiments were performed on a micrOTOF Bruker (electrospray ionization ESI^+^, 50–1000 in low and 50–2500 in width).

The setup and the protocol to evaluate the photophysical characterizations including the absorption, fluorescence, and singlet oxygen emission spectra in addition to the fluorescence and singlet oxygen lifetimes have been previously described by our team [71]. The absorption spectra were recorded on a UV-3600 UV–vis double beam spectrophotometer (Shimadzu, Marne La Vallée, France) in the wavelength range of 200-800 nm. The measurements were performed on the molecules dispersed in ethanol in quartz cuvettes with an optical path of 10 mm. The fluorescence spectra were recorded on a Horiba Jobin Yvon Fluorolog-3 (FL3-222) spectrofluorimeter equipped with a 450W xenon lamp, a thermostated cell compartment (25 °C), a R928 (Hamamatsu, Massy, France) UV–vis photomultiplier and a liquid nitrogen cooled InGaAs infrared detector (DSS-16A020L Electro-Optical System Inc., Phoenixville, PA, USA). The excitation spectrometer used is a SPEX double grating monochromator (1200 grooves per mm blazed at 330 nm). The fluorescence was measured using the UV–vis detector through a SPEX double grating monochromator (600 grooves per mm blazed at 500 nm). The singlet oxygen production was measured using the IR detector through a SPEX double grating monochromator (600 grooves per mm blazed at 1 μm).

Fluorescence quantum yields (Φ_f_) were determined using a tetraphenyl porphyrin (TPP) solution as the fluorescence standard (Φ_f_ = 0.11 in toluene, taking into account solvent refractive index and absorption efficiencies [72]. For the direct determination of ^1^O_2_ quantum yield (Φ_Δ_), excitation was performed with a Xe-arc, the light was separated in a SPEX 1680, 0.22 µm double monochromator. Detection at 1270 nm was carried out through a PTI S/N 1565 monochromator and the emission was monitored by a liquid nitrogen cooled Ge-detector (EO-817L, North Coast Scientific Co, OH, USA). Rose Bengal (RB) was chosen as a reference solution because of its high ^1^O_2_ quantum yield in EtOH (Φ_Δ_ = 0.68) [73]. The absorbance value at the excitation wavelengths (412 nm) of both the reference and the sample solutions were set to approximately 0.2 by dilution. The samples were all prepared in ethanol.

The quantum yield of fluorescence is determined by the equation (1):

(1) $$\Phi_{f}=\Phi_{f0}\times \frac{I_{f}}{I_{f0}}\times \frac{\mathrm{DO}}{{\mathrm{DO}}_{0}}\times {\left(\frac{n}{n_{0}}\right)}^{2}$$

where Φ_f_ and Φ_f0_, If and If_0_, DO and DO_0_, n and n_0_ are the quantum yields, intensities, optical densities and refraction indices of the sample and reference, respectively.

The quantum yield of singlet oxygen production is determined by the equation (2):

(2) $$\Phi_{\Delta }=\Phi_{\Delta 0}\times \frac{I}{I_{0}}\times \frac{\mathrm{DO}}{{\mathrm{DO}}_{0}}$$

where Φ_Δ_ and Φ_Δ0_, I and I_0_, DO and DO_0_ are the luminescence quantum yields of singlet oxygen, the luminescence intensities and the optical densities of the sample and reference, respectively.

Fluorescence lifetimes were measured by Time-Correlated Single Photon Counting (TCSPC) using for excitation a pulsed laser diode LDH-P-C-405 coupled with a PDL 800-D driver (both from PicoQuant GmbH, Berlin, Germany) emitting at 408 nm (FWHM < 70 ps, 1 MHz) and an SPCM-AQR-15 avalanche photodiode (EG & G, Vaudreuil, Canada) coupled with a 650 nm (±10 nm) interference long-wave pass filter as the detection system. The acquisition system was a PicoHarp 300 module with a PHR-800 four-channel router (both from PicoQuant GmbH, Berlin, Germany). Fluorescence decays were recorded using the single photon counting method (four optical faced quartz cells for pure products in ethanol). Data were collected, and up to 1000 counts were accumulated in the maximum channel and analyzed using Fluofit TCSPC software (PicoQuant GmbH, Berlin, Germany) based on iterative reconvolution using a Levensberg-Marquandt algorithm. Singlet oxygen lifetime measurements have been performed on a TEMPRO-01 spectrophotometer (Horiba Jobin Yvon, Palaiseau, France). The apparatus was composed of a pulsed diode excitation source SpectraLED-415 emitting at 415 nm, a cuvette compartment, a Seya–Namioka type emission monochromator (600–2000 nm), and an H10330-45 near-infrared photomultiplier tube with a thermoelectric cooler (Hamamatsu, Massy, France) as the detection system. The system was monitored by a single photon counting controller FluoroHub-B and the software DataStation and DAS6 (Horiba Jobin Yvon, Palaiseau, France).

### 2.6. Estimation of the PS or Peptide Loading

The loading of the PS onto the AuNRs was estimated relying on the molar extinction coefficient (ε) calculated for the MI-K(Pyro)DKPPR-OH conjugate. A calibration curve was developed based on the different concentrations of the conjugate, in water, that correspond to different optical densities (OD). The ε was determined using Beer Lambert’s Law at 381 nm, which constitutes the wavelength of the Soret band of Pyro in water.

(3) A = εLC

where A, *ε*, L and C stand for the absorption (OD), molar absorptivity (extinction, L·mol^−1^·cm^−1^), length (cm) and concentration (mol·L^−1^).

The concentration of Pyro in AuNRs@PEG-MI-K(Pyro)DKPPR-OH was calculated for a mass concentration of 1 mg·mL^−1^. Moreover, the concentration of the peptide was also determined, as only 1 peptide unit is conjugated to 1 PS molecules. On this basis, the amount of peptide was also estimated to 1 mg·mL^−1^ of the functionalized AuNRs.

### 2.7. Affinity to NPR-1

The affinity of the compounds for NRP-1 was realized as previously described in terms of IC50 in a competition study [53]. Briefly, the surface of the high protein-binding capacity polystyrene 96 well ELISA plate (NUNC MaxiSorp™, Sigma-Aldrich, Saint-Quentin Fallavier, France) was coated with NRP-1 at a concentration of 2 μg·mL^−1^ in PBS and kept overnight at room temperature. The plate was then blocked with a solution of PBS containing 0.5 % bovine serum albumin (BSA) during 1 h at 37 °C, to inhibit non-specific interactions. Binding of the tested compounds to NRP-1 was evaluated using 5 ng·mL^−1^ of biotinylated VEGF-A165 in blocking buffer containing 2 μg·mL^−1^ of heparin. Biotinylated VEGF-A165 was added to the coated wells, in competition, or not, with different peptide concentrations of MI-K(Pyro)DKPPR-OH, functionalized AuNRs (AuNRs@PEG-MI-K(Pyro)DKPPR-OH) or H-KDKPPR-OH peptide alone and non-labeled VEGF-A165, as a positive control. The plates were further incubated for 2 h at room temperature. After that, the plates were washed 3 times and the amount of bound biotinylated VEGF-A165 was assayed, as it was stained with streptavidin horseradish peroxidase conjugate and its substrate (tetramethylbenzidine and H_2_O_2_). After 20 min of incubation at room temperature, the reaction was stopped by the addition of 2N sulfuric acid. The corresponding optical densities (OD) were then measured at a wavelength of 450 nm. The obtained results were expressed as relative absorbance to wells containing only blocking buffer. For this assay, three wells were used per condition [53,54].

### 2.8. In Vitro Studies

#### 2.8.1. Cell Culture Conditions

For in vitro experiments, U87 glioblastoma cells (ATCC^®^ HTB-14™) were cultivated in Dulbecco’s modified eagle’s medium (DMEM) supplemented with sodium pyruvate 1.5 mM, MEM AA, MEM NE AA, MEM vitamins, *l*-Ser 14 μg·mL^−1^, *l*-asp 25 μg·mL^−1^, *l*-Glu 2.5 mM, 100 U·mL^−1^ penicillin, 100 μg·mL^−1^ streptomycin, and 20% FCS. They were cultured under standard cell culture conditions while incubated in 5% CO_2_/80% humidified air at 37 °C.

#### 2.8.2. Photodynamic Activity

Cell survival of U87 after incubation with the hybrid AuNRs, the PS-peptide conjugate or the free PS in the dark (cytotoxicity) or after light exposure (phototoxicity) was investigated using an MTT assay as previously described [74]. Briefly, U87 cells were plated into 48-well plates at an initial cell density of 2 × 10^4^ cells/cm^2^ in culture medium, incubated at 37 °C during 48 h. Old culture media were discarded and the wells were filled with 250 µL of culture medium containing different concentrations of the tested molecules (AuNRs@PEG-MI-K(Pyro)DKPPR-OH, MI-K(Pyro)DKPPR-OH and Pyro) at a final PS concentration of 10, 20, and 30 μM. After 24 h, the wells were washed three times with HBSS and filled with culture medium. To evaluate the cytotoxicity of the tested molecules towards the U87 cells, one plate was kept incubated in the dark. However, the phototoxicity induced by those molecules was assessed by exposing the cells to light at a fluence of 10 J·cm^−2^ (fluence rate 4.54 mW·cm^−2^, duration: 36 min 42 s) using a 652 nm diode laser system (Ceralas, Biolytec, Jena, Germany). The cells were further incubated for 24 h. The cell survival was measured by MTT assay where the cells were incubated for 3 h at 37 °C with 0.4 mg·mL^−1^. After that, the excess of MTT was discarded and the formazan crystals that developed in the living cells were dissolved by DMSO. The absorption of formazan was measured at 540 nm with a background subtraction at 630 nm using a Multiskan Ascent plate reader (Thermo Scientific, Vantaa, Finland). Experiments were carried out in triplicates.

### 2.9. Statistical Analysis

All in vitro experiments were realized in triplicate and expressed as a percentage of variation from control cells. The results were given as mean ± standard deviation (SD). Differences among groups were evaluated using variance analysis (Anova) followed by Bonferroni test (GraphPad Prism 6.0 software, San Diego, CA, USA). A value of *p* < 0.05 was considered as statistically significant. * Versus control cells.

## 3. Results and Discussion

### 3.1. Synthesis and Characterization of the AuNRs@PEG

**Scheme 1:**
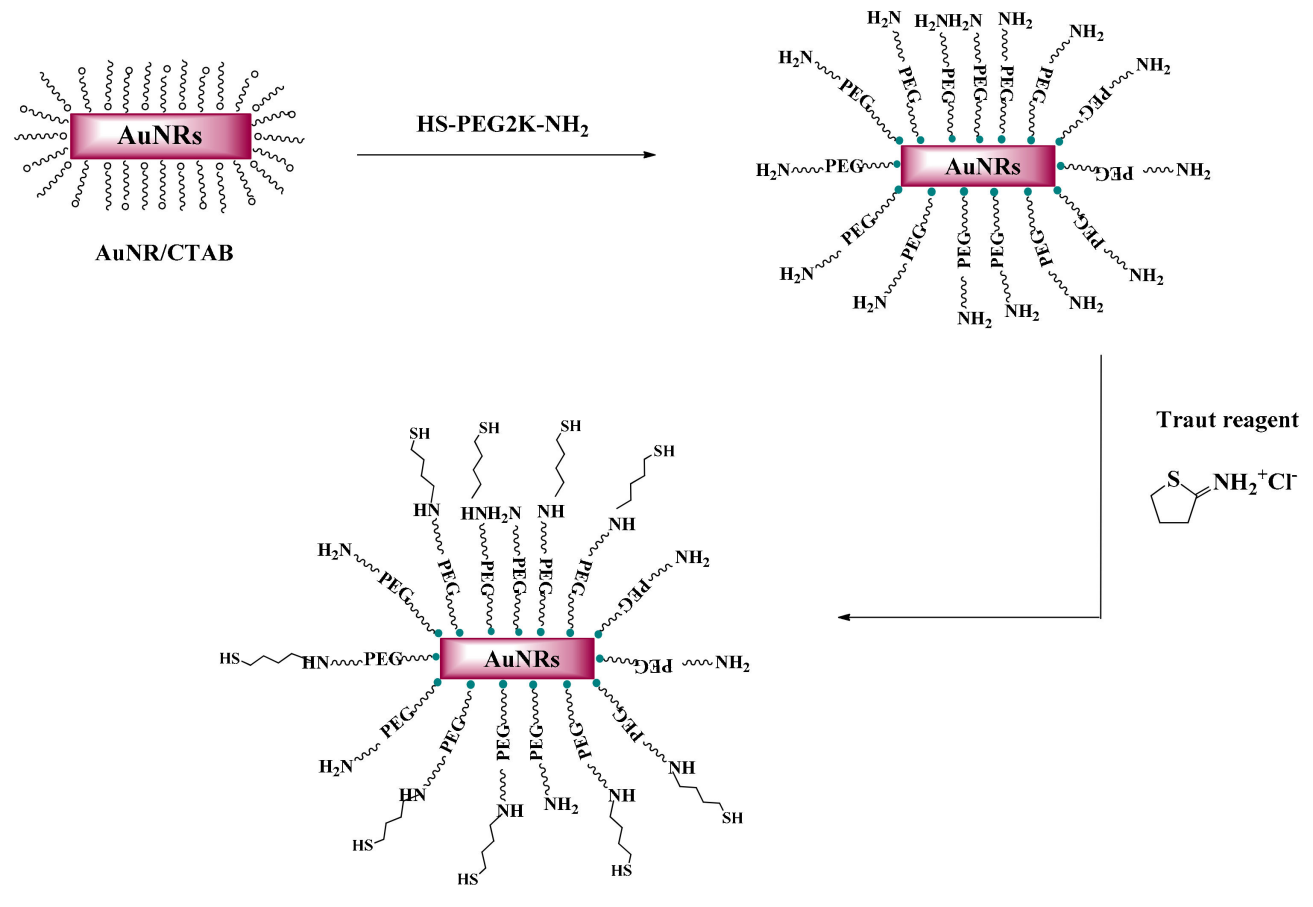
The synthetic pathway of gold nanorods (AuNRs)@ polyethylene glycol (PEG).

The synthesis and the functionalization of AuNRs have been thoroughly described in the literature [46,49,75]. In our current work, the CTAB-coated AuNRs were prepared by the seed-mediated method. Then, the AuNRs were conjugated with PEG in order to effectively diminish the cytotoxicity that arises from the presence of CTAB and silver ions in the synthesis (Scheme 1) [49].

The quantity of PEG per AuNRs was investigated using fluorescamine- and ninhydrin-based assays. The corresponding calibration curves are displayed in the Supporting Information, Figure S1 and Figure S2, respectively, in addition to the table that shows the concentrations in the initial and the supernatant solutions (Table S1). Table 1 shows the results obtained in both assays.

**Figure 1:**
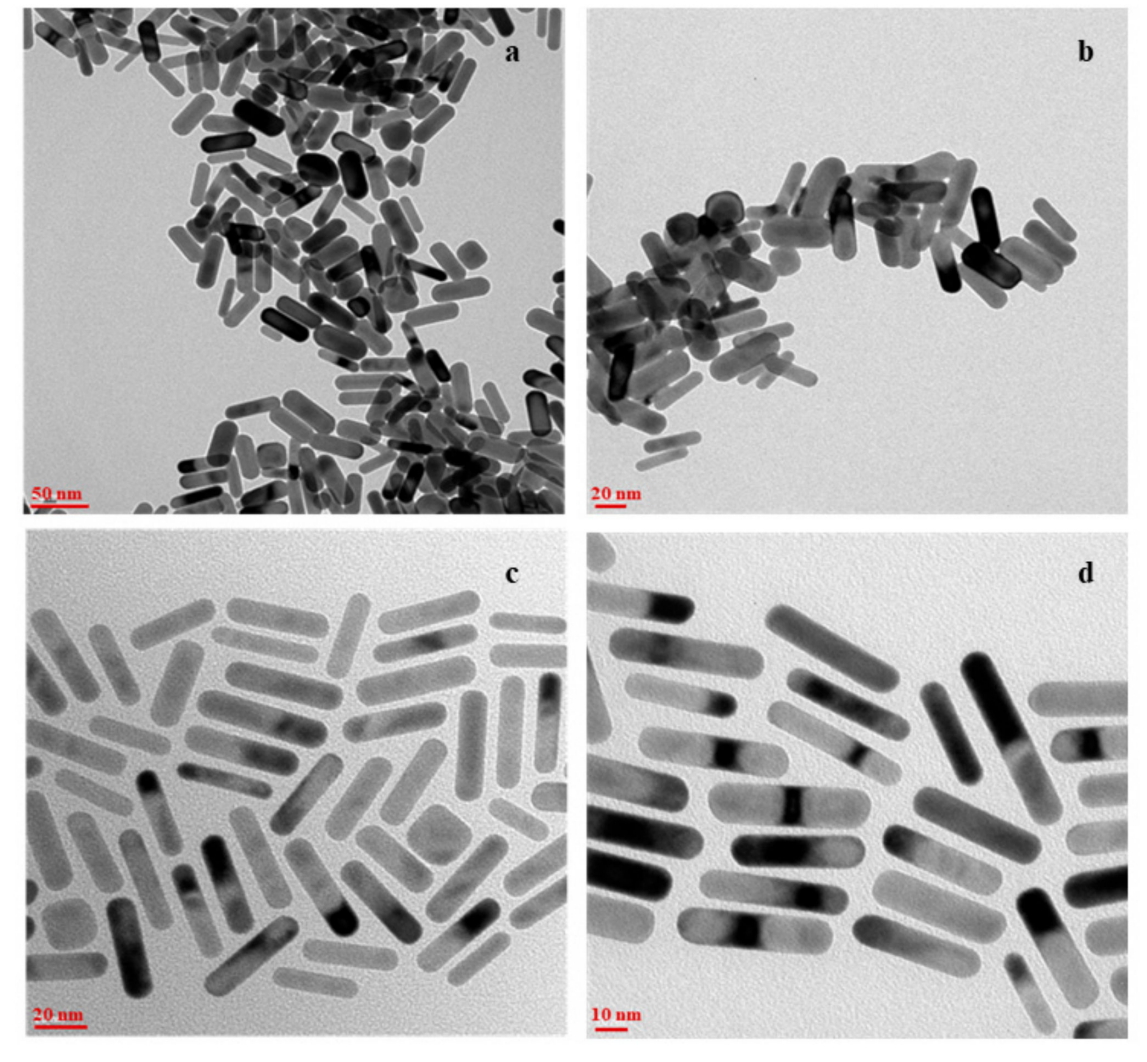
The TEM images of (**a**,**b**) AuNRs@CTAB and (**c**,**d**) AuNRs@PEG.

The TEM images of the AuNRs before (CTAB-capped) (Figure 1a,b) and after PEGylation (Figure 1c,d) are shown in Figure 1. The comparison between both images at the same scale, 20 nm, confirms that the dispersion of the nanorods is, as expected, much better after PEGylation and therefore the colloidal stability becomes greater (Figure 1c,d). This result is more demonstrated by observing the TEM image of the AuNRs@PEG at a smaller scale, 10 nm (Figure 1d). The average length of the nanorods is about 44 ± 4 nm and the average width is about 8 ± 2 nm.

**Figure 2:**
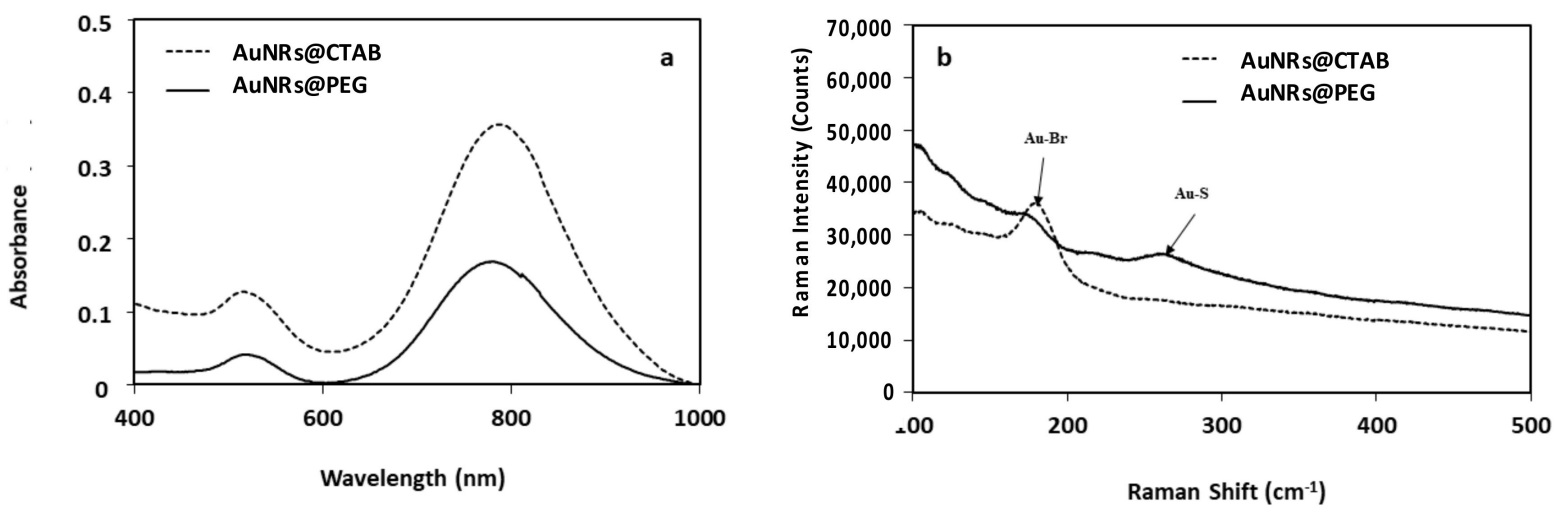
(**a**) Absorption spectra of AuNRs before and after PEGylation by HS-PEG2K-NH_2_, (**b**) Raman spectra of AuNRs and AuNRs@PEG.

Figure 2a displays the UV-vis absorption spectra of the AuNRs before and after PEGylation in water. By observing both spectra, we can confirm that the PEGylation kept the absorption profile of the AuNRs intact with an absorption maximum localized in the near infrared region at a wavelength of about 800 nm [76]. This profile consists of two absorption bands, the longitudinal (LPB) and the transverse plasmon bands (TPB), that are attributed, respectively, to the oscillation of the electron along the long and the short axes of the AuNRs [77].

The Raman spectra of the AuNRs prior to and after the surface modification by PEG in the range between 100 and 500 cm^−1^ are revealed in Figure 2b. For the AuNRs@CTAB, only one peak can be observed at 180 cm^−1^. This peak is attributed to the Au-Br bond. However, in the same range, the Raman spectrum of the AuNRs@PEG shows a new peak at 258 cm^−1^ which is assigned to the Au-S bond. These outcomes suggest that the PEGylation occurred and the great decrease in the intensity of the peak corresponding to Au-Br means that CTAB has been substituted by PEG [49].

The zeta potential values are also investigated. In addition to the Raman spectra, the change in the surface charge from −7.59 mV in the case of AuNRs@CTAB to +0.53 mV for the AuNRs@PEG constitutes the most relevant result for proving the presence of PEG in the organic shell of the nanorods.

**Figure 3:**
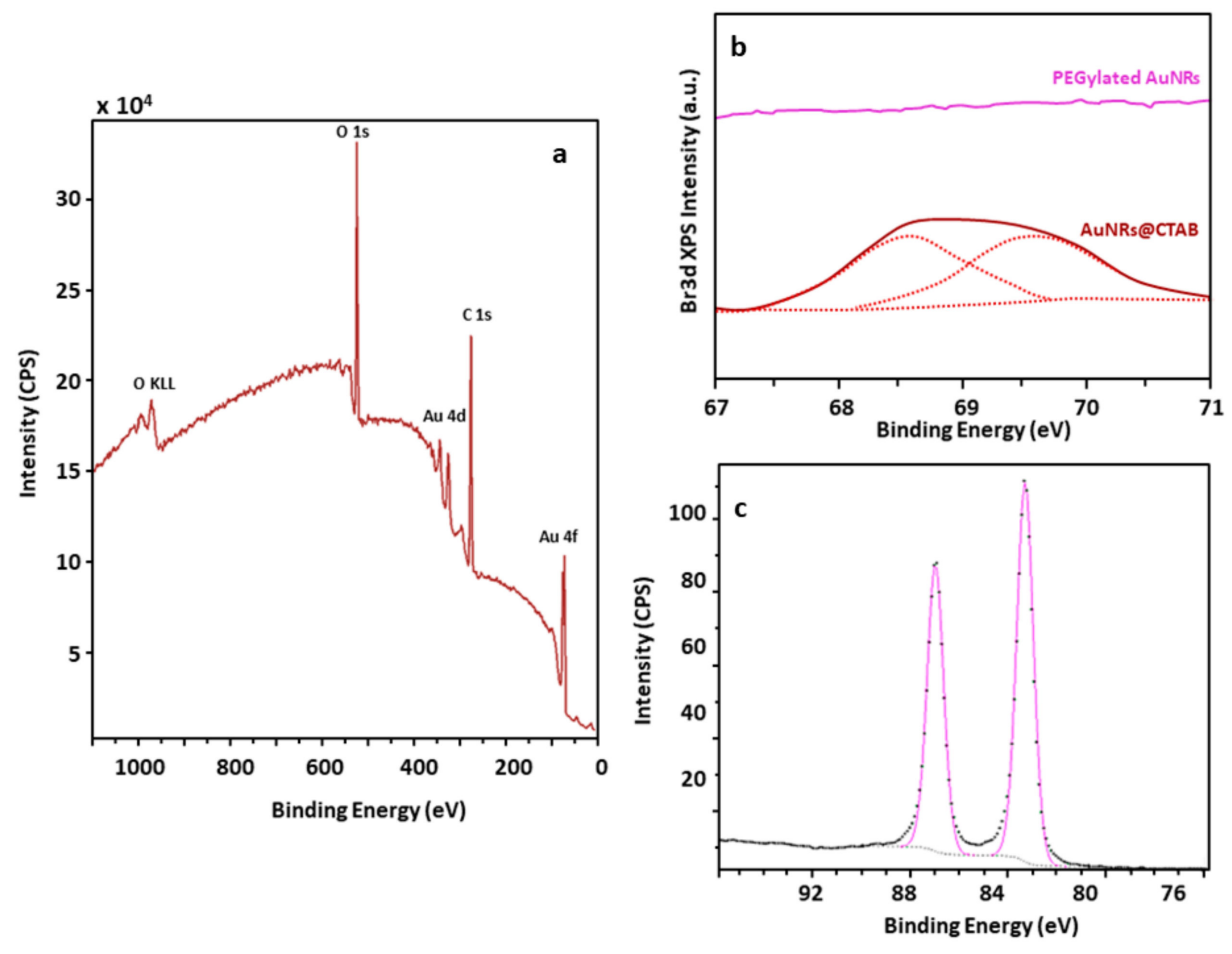
(**a**) wide scan XPS spectrum of the AuNRs@PEG, (**b**) high-resolution Br 3d XPS spectra of AuNRs@PEG and AuNRs@CTAB, (**c**) high-resolution spectra of the Au 4f lines for the AuNRs@PEG.

The XPS characterization technique was used to explore and approve the chemical state of the AuNRs@PEG and the resulting data is displayed in Figure 3. Figure 3a reveals the wide scan XPS spectrum of the AuNRs@PEG, which shows the presence of several peaks that correspond well with the carbon, oxygen and gold core levels. The high-resolution Br 3d XPS spectrum (Figure 3b) of the AuNRs@PEG demonstrates the absence of the peaks corresponding to Br 3d (68.6 and 69.6 eV) in contrary to that obtained for AuNRs@CTAB. The attained results postulate that the PEGylation was effectual and the used synthesis method was successfully and completely capable of eliminating CTAB molecules from the synthesized AuNRs [49,78]. The high-resolution spectra of the Au 4f lines (Figure 3c) reveals one doublet localized at 83.2 and 87.1 eV, attributed, respectively, to Au 4f_7/2_ and Au 4f_5/2_. The detection of Au 4f_7/2_ signal at 83.2 eV indicates the presence of Au in its metallic reduced form Au(^0^) [35,79,80].

### 3.2. Synthesis and Characterization of Peptide Derivatives and Hybrid AuNRs

**Scheme 2:**
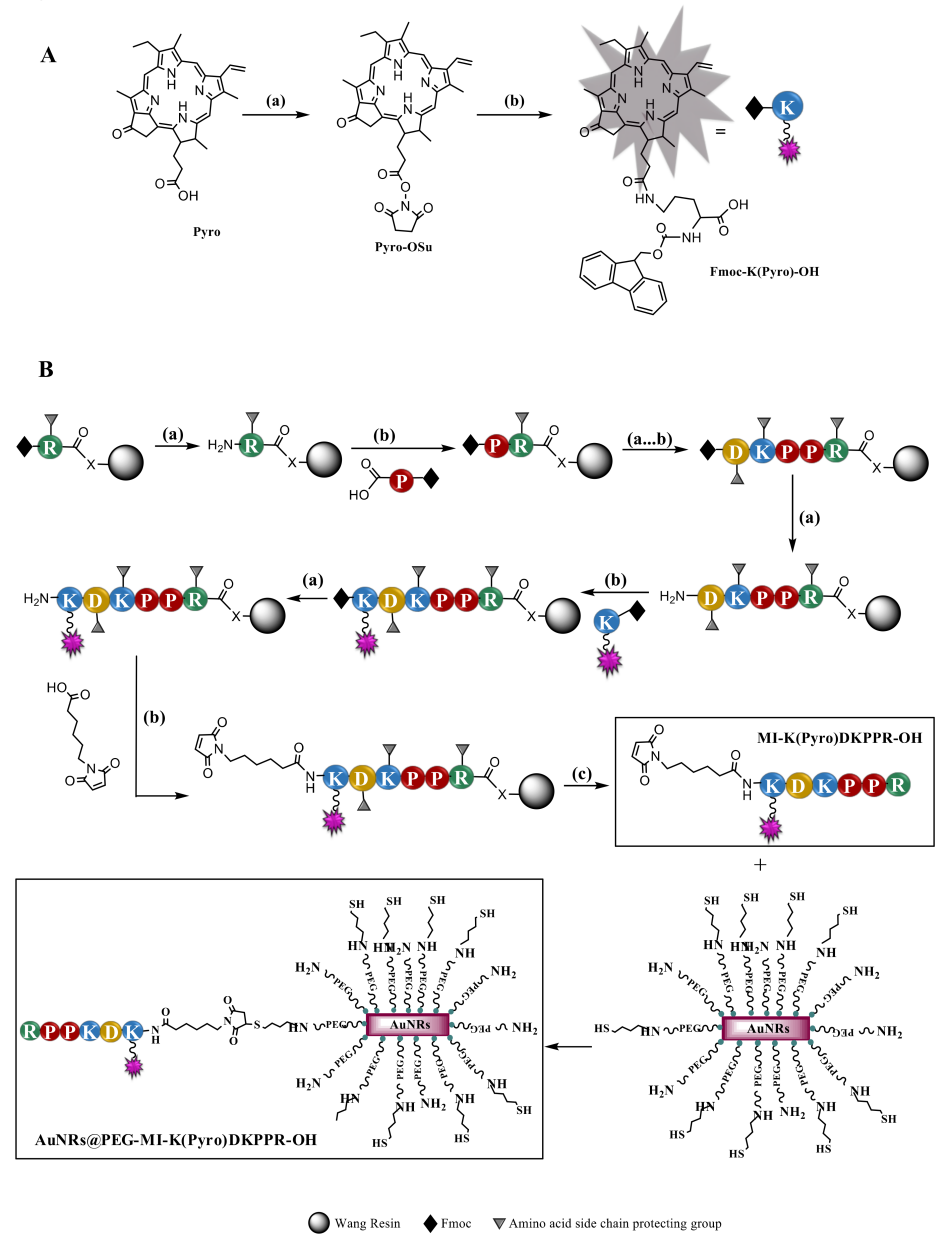
The synthetic pathway of AuNRs@PEG-MI-K(Pyro)DKPPR-OH. (**A**) Preparation of Fmoc-K(Pyro)-OH: (a) HOSu, EDCI, DCM, 45 °C, overnight and (b) Fmoc-K-OH, HCl, Et_3_N, CHCl_3_, DMF, 0 °C to RT, 24 h. (**B**) Solid-phase synthesis of the MI-K(Pyro)DKPPR-OH compound: (a) *N*-deprotection: 25% piperidine in DMF, (b) coupling: HBTU/NMM and (c) cleavage: TFA/TIPS/ H_2_O (92.5/2.5/5) and finally the coupling of the peptide to the AuNRs via click-chemistry. Abbreviations: Photosensitizer (PS); dichloromethane (DCM); room temperature (RT); *N*-methylmorpholine (NMM); *N*-methylpyrrolidinone (NMP); trifluoroacetic acid (TFA); triisopropylsilane (TIPS); 1-ethyl-3-(3-dimethylaminopropyl)carbodiimide (EDCI); dimethyl formamide (DMF); *N*-Hydroxysuccinimide (HOSu); *N,N,N′,N′*-Tetramethyl-*O*-(1*H*-benzotriazol-1-yl)uronium hexafluorophosphate (HBTU).

The AuNRs@PEG were further functionalized as destined to be used for the targeting of the NRP-1 recombinant protein and for anti-cancerous PDT application. In action to serve this goal while maintaining the affinity of H-KDKPPR-OH, our team developed a certain approach consisting of (a) grafting the PS onto a Lysine residue, (b) building up the modified peptide PP-PS, and (c) functionalizing the AuNRs with PP-PS. The PS (Pyro in our case) is coupled in the form of Fmoc-K(Pyro)-OH to the H-DKPPR-Wang growing peptide by classical Fmoc/tBu solid-phase synthesis, affording PP-PS (Scheme 2). This PP-PS conjugate was then grafted onto the surface of the nanorods through another click-chemistry reaction well-known as maleimido-thiol reaction [53]. The maleimide group reacts specifically with sulfhydryl groups when the pH of the reaction mixture is between 6.5 and 7.5; the result is the formation of a stable thioether linkage that is not reversible. Based on this synthetic approach we produced the AuNRs@PEG-MI-K(Pyro)-DKPPR-OH (Scheme 2). As mentioned above, the PS-Peptide conjugate, MI-K(Pyro)DKPPR-OH, was synthesized by the classical Fmoc/tBu solid-phase peptide synthesis. All the steps of this synthesis are depicted in Scheme 2. In this procedure, we used lysine amino acid since it has two amine groups to link both the PS and the maleimide and a carboxylic group to be coupled to the *N*-terminal of the growing peptide. Fmoc-K(Pyro)-OH was synthesized in liquid phase by coupling Pyro-OSu to the ε-amino group of the side chain of the Fmoc-Lys-OH (See characterizations of Pyro-OSu and Fmoc-Lys(Pyro)-OH in Figures S3–S6.). Fmoc-K(Pyro)-OH was then coupled to the H-DKPPR-OH peptide linked to the wang resin in a typical Fmoc/tBu solid-phase synthesis followed by the deprotection of the amine group of lysine to allow the coupling of the maleimidohexanoic acid. MI-K(Pyro)DKPPR-OH peptide was cleaved from the resin with a mixture of TFA, TIPS and water (92.5/2.5/5), precipitated with cold ether and washed several times to be recuperated by centrifugation. The filtrate was dried under vacuum and freeze dried. The structures of the synthesized peptides were confirmed by high-resolution mass spectrometry (HRMS) and ^1^H NMR (Figures S7–S12 and Table S2–S4).

#### 3.2.1. Photophysical Properties

The photophysical properties at the different synthetic stages of the hybrid AuNRs@PEG-MI-K(Pyro)DKPPR-OH, including the peptide derivatives as intermediates, were studied and compared to that of free Pyro. These properties include the UV-vis absorption, the florescence emission (quantum yield (Φ_f_) and lifetime (τ_f_)) and the ^1^O_2_ production (quantum yield (Φ_Δ_) and lifetime (τ_Δ_)). To determine these properties, the optical density (OD) of the Soret band of the Pyro component (412 nm) was adjusted at about 0.2. Controls, such as TPP dissolved in toluene, as a standard for fluorescence emission, and RB dissolved in ethanol, as a standard for ^1^O_2_ production, were used. Ethanol was chosen to dissolve the studied molecules since it is a convenient solvent for the detection of fluorescence and ^1^O_2_ emission. The excitation wavelength for the emission and the lifetime measurements is 412 nm.

**Figure 4:**
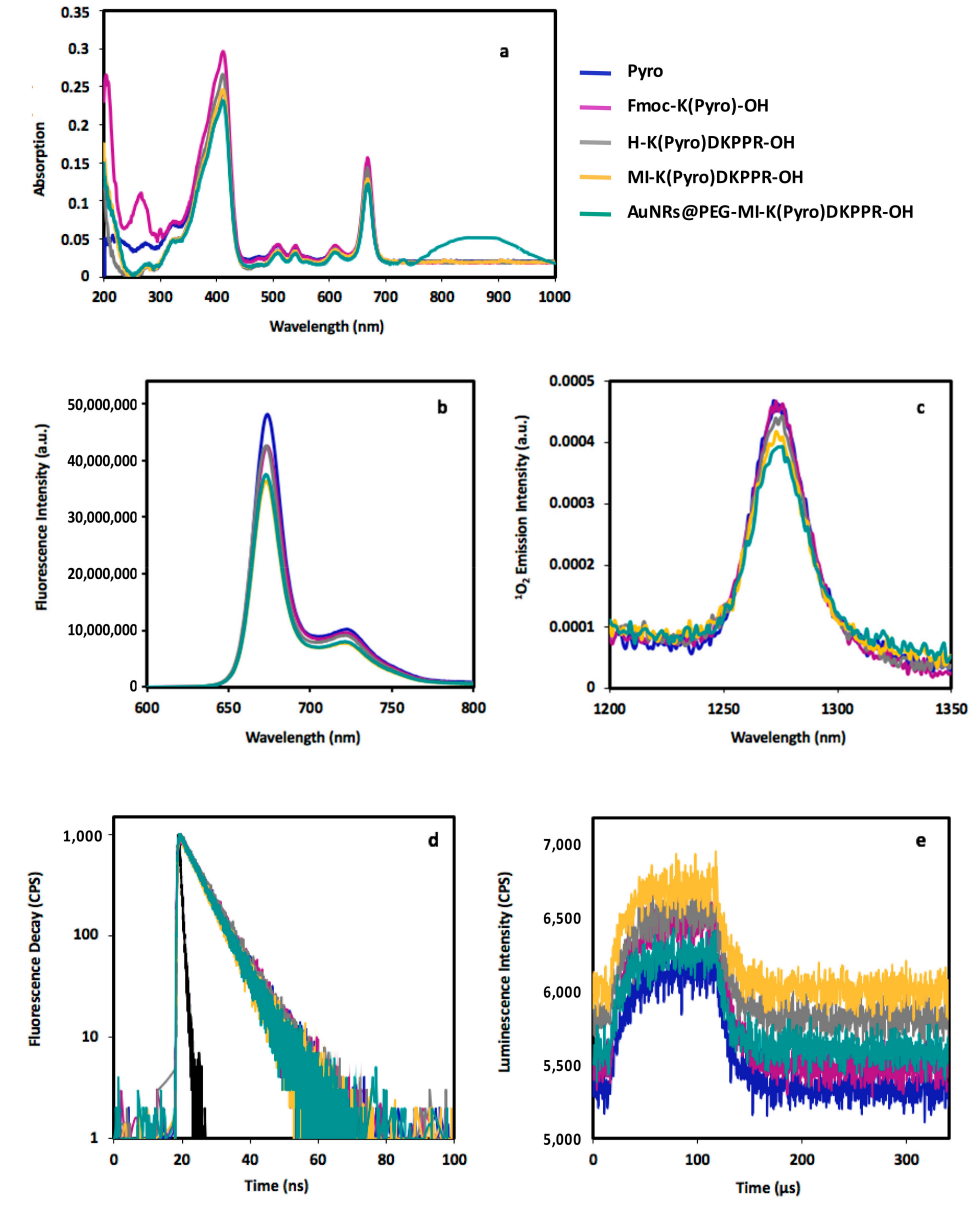
The photophysical characterizations including (**a**) UV-vis absorption spectra, (**b**) fluorescence and (**c**) singlet oxygen emissions, (**d**) fluorescence and (e) singlet oxygen decays of the functionalized gold nanorods (AuNRs) and the different intermediates studied in ethanol at λ_exc_ = 412 nm.

By observing Figure 4a, it appears that the UV-vis absorption spectrum of Pyro in ethanol stayed intact upon all the modifications, Fmoc-K(Pyro)-OH, H-K(Pyro)DKPPR-OH and MI-K(Pyro)DKPPR-OH. Most importantly, after grafting MI-K(Pyro)DKPPR-OH onto the AuNRs, the absorption spectrum of the elaborated AuNRs@PEG-MI-K(Pyro)DKPPR-OH, in ethanol, preserved the characteristic peaks of Pyro. These include a Soret band localized at 412 nm and four Q-bands between 450 and 750 nm. Thus, the photoresponse of the sensitized-AuNRs is modified as it encompasses that of the Pyro after the chemical click of the peptide- Pyro conjugate onto the surface of the former. This implies that the synthetic pathway appears to be successful.

Figure 4b and 4d display the florescence emission and decay of all the studied molecules dispersed in ethanol. It can be clearly observed that Fmoc-K(Pyro)-OH, H-K(Pyro)DKPPR-OH, MI-K(Pyro)DKPPR-OH as well as the produced AuNRs@PEG-MI-K(Pyro)DKPPR-OH exhibit fluorescence while maintaining the same shape of that exposed by free Pyro. Similar fluorescence quantum yields (Φ_f_) that lie in the range between 0.29 and 0.38 are also recorded. In addition, the fluorescence lifetimes (τ_f_) are detected to be similar for the free Pyro, that is coupled to the peptides and the final hybrid nanorods dispersed in ethanol. The calculated fluorescence lifetimes range between 6.5 and 6.9 ns using a single exponential to fit the data (Figure 4d and Table 2).

These findings are also consistent with the Φ_f_ value of Pyro reported by other authors (Φ_f_ = 0.3 and in methanol) [16]. These results emphasize that the coupling of Pyro to the peptides and the grafting of the PP-PS conjugate to the AuNRs did not alter the fluorescence emission properties of Pyro. Furthermore, it should be noted that the hybrid AuNRs, possess an important absorption Q-band and 670 nm which is the characteristic peak of the Pyro PS, this means that the AuNRs absorb in the near-infrared (NIR) region and can exhibit fluorescence for tumor imaging. This property can serve in the biomedical applications that necessitate a higher degree of penetration of the NIR light in deep tissues [81].

Similarly, to the fluorescence, we compared, in ethanol, the luminescence emission at 1270 nm induced by the excitation at 412 nm of all the compounds. The ^1^O_2_ quantum yields of the PP-PS conjugates and the hybridized AuNRs lie in the range of 0.39 to 0.51 (Figure 4c and Table 2). These values imply that the ^1^O_2_ quantum yield of free Pyro (Φ_Δ_ = 0.5), is scarcely affected by the conjugation. As for the ^1^O_2_ lifetimes, Figure 4e reveals the decay curves. These curves are constituted of three parts as they start by the augmentation of the luminescence that evolves into a plateau and ends by the decay of ^1^O_2_ luminescence. After fitting the decay curves, we can extract the ^1^O_2_ lifetimes. The obtained lifetimes are similar to all the studied molecules and the hybrid AuNRs that ranges between 12 and 14 μs (Table 2). It may be concluded that the ^1^O_2_ does not interact with the PS [74,82].

#### 3.2.2. Estimation of Pyro Concentration in the MI-K(Pyro)DKPPR-OH and Hybridized AuNRs

The loading of the PS onto the AuNRs was estimated relying on the molar extinction coefficient (ε) calculated for the MI-K(Pyro)DKPPR-OH conjugate in water at 381 nm. The ε extracted from the calibration curve is 64012 L·mol^−1^·cm^−1^. On this basis, the absorption of hybridized AuNRs at a known mass concentration (mg·mL^-1^) was recorded at the same wavelength. By taking into consideration the value of molar extinction, the molecular weight of Pyro and the dilution factors, 1 mg·mL^−1^ of these AuNRs holds 257.7 µM of PS and correspondingly of peptide, since 1 peptide is conjugated to 1 PS.

### 3.3. Affinity to Recombinant NRP-1 Protein

**Figure 5:**
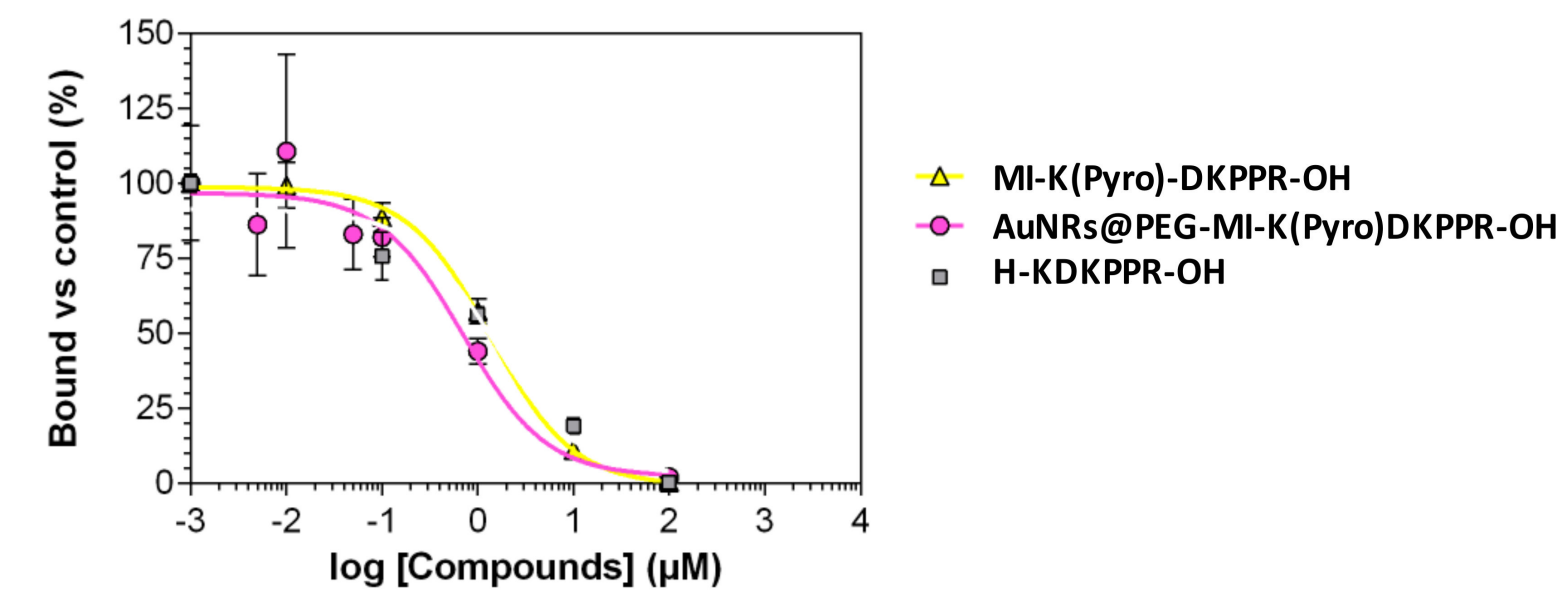
Binding of H-KDKPPR-OH, AuNRs@PEG-MI-K(Pyro)DKPPR-OH, and MI-K(Pyro)DKPPR-OH to recombinant NRP-1 protein.

The molecular affinity of compounds for the recombinant NRP-1 protein was evaluated in terms of the half maximal inhibitory concentration (IC_50_ values) through a competitive binding assay with biotinylated VEGF165 and different concentrations of “KDKPPR” either free, conjugated with the PS or grafted onto AuNRs. The results are displayed in Figure 5.

Free H-KDKPPR-OH peptide succeeds to displace the binding of the biotinylated VEGF165 onto the receptor with an IC_50_ = 2 µM [53]. This competitive binding study reveals that the conjugated MI-K(Pyro)DKPPR-OH peptide possesses a molecular affinity as good as that of free H-KDKPPR-OH with an inhibition concentration, IC_50_, of 1.5 µM. More importantly, the binding of biotinylated VEGF165 for the recombinant NRP-1 protein was effectively dislodged by the novel functionalized AuNRs (AuNRs@PEG-MI-K(Pyro)DKPPR-OH) synthesized in this study, with an IC_50_ value of 1.1 µM. This emphasizes that grafting the peptide conjugate onto the AuNRs does not jeopardize the capability of the latter to bind to NRP-1. These outcomes illustrate the good capacity of the hybrid AuNRs for the active targeting of NRP-1, in addition to the passive targeting usually achieved in vivo through enhanced permeation and retention (EPR) effect thanks to the nanosize of the particles [83,84,85].

### 3.4. Photodynamic Activity

#### 3.4.1. Cytotoxicity

**Figure 6:**
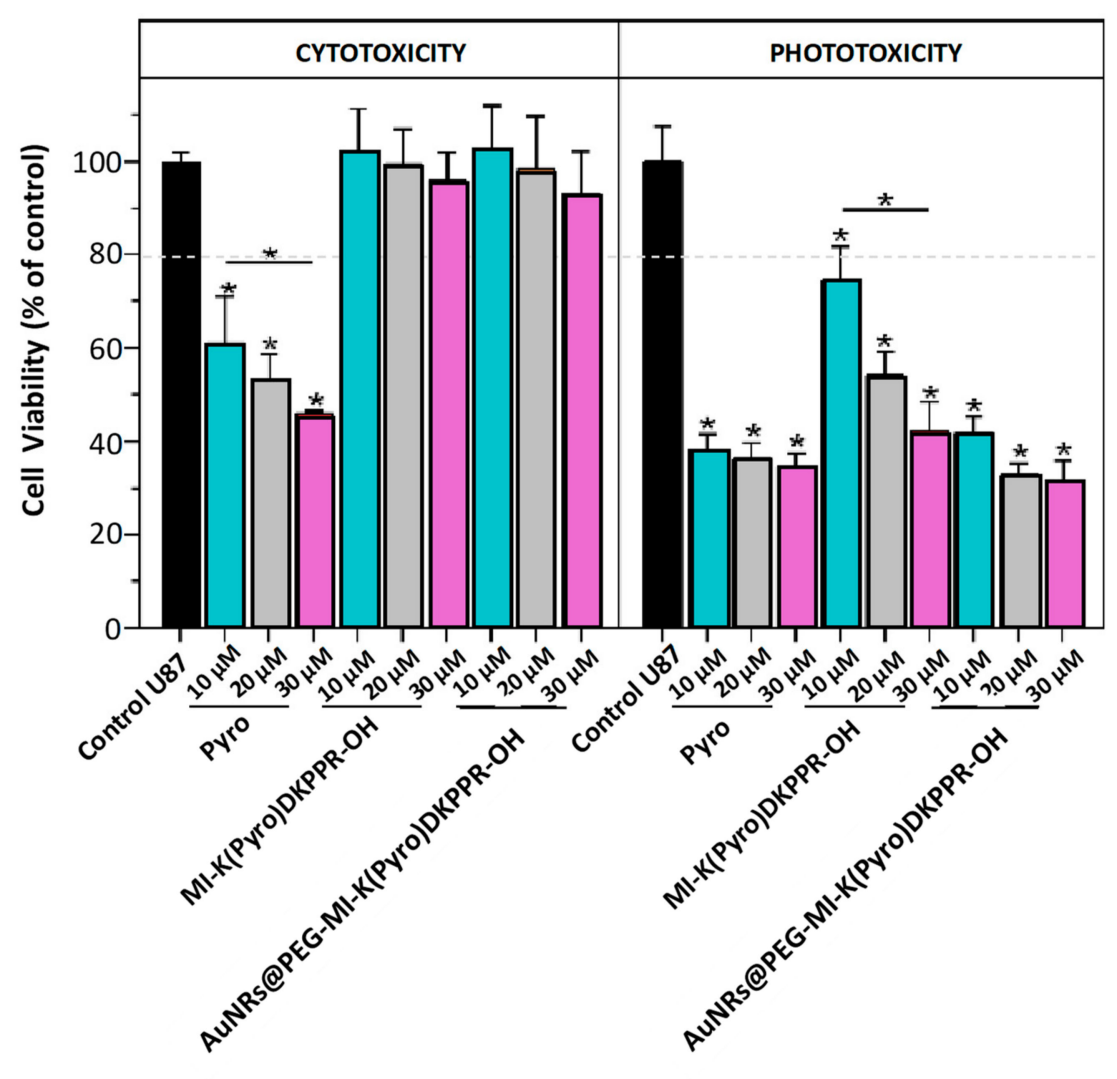
Cell viability of U87 cells treated with free Pyro, MI-K(Pyro)DKPPR-OH conjugate and AuNRs@MI-K(Pyro)DKPPR-OH incubated into the dark (Cytotoxicity) or after irradiation with a light dose of 10 J/cm2 (Phototoxicity). Results were expressed as percentage of values obtained from untreated cells and given by mean ± SD, *n* = 3, *p* < 0.05. * Versus control cells.

The MTT assay was performed to evaluate the cytotoxicity of the tested PS, PP-PS conjugate and hybrid AuNRs on U87 cells in vitro. The cells were exposed to free Pyro, MI-K(Pyro)DKPPR-OH conjugate and AuNRs@MI-K(Pyro)DKPPR-OH at PS concentrations of 10, 20 and 30 µM in DMEM during 24 h in the dark. Results given in Figure 6 show that Pyro decrease the metabolic activity of U87 cells even at the lowest concentration used (10 µM). Nevertheless, both the peptide-PS conjugate and the hybrid AuNRs revealed no cytotoxic effect even at a PS concentration of 30 µM. It appears that coupling the PS to the peptide and grafting this conjugate onto the AuNRs diminished the dark toxicity displayed by the high concentrations of the PS. These results also highlight on the efficiency of conjugating the AuNRs with PEG in terms of decreasing and even terminating their cytotoxicity that normally arises from the CTAB and silver ions employed in their fabrication.

#### 3.4.2. Phototoxicity

After validating the good potential of the hybrid AuNRs in the active targeting of the NRP-1 protein and the lack of any dark cytotoxicity even at high PS concentrations, further phototoxicity assays were performed. To estimate the efficiency of hybrid AuNPs on glioma cells, in vitro experiments were conducted on U87 cells treated during 24 h with hybrid AuNRs at increasing PS concentrations (10, 20 and 30 µM), as compared to MI-K(Pyro)DKPPR-OH and free Pyro at the same PS dose. The results of the cell viability after light exposure (652 nm, 10 J/cm^2^) are shown in Figure 6. In fact, the low PDT efficacy that we have obtained with free Pyro can be explained by the aggregation of the PS. Since Pyro, similarly to common PSs, is poorly soluble in biological media, and thus it tends to aggregate easily mediating a self-quenching that causes the modification of the photophysical characteristics especially the decrease of singlet oxygen responsible for the cell killing [3,4,14,86]. The functionalized AuNRs decrease the viability of U87 cells by 58, 65, and 67% when exposed to the AuNRs at PS dose of 10, 20, and 30 µM, respectively. Moreover, the MI-K(Pyro)DKPPR-OH conjugate decrease the viability of cells by 25, 46 and 58% when exposed to 10, 20, and 30 µM of conjugate respectively. This indicates a lower photodynamic activity of MI-K(Pyro)DKPPR-OH conjugate compared to functionalized AuNRs. On this basis, it is obvious that coupling this PP-PS conjugate to the AuNRs enhanced the photodynamic activity of the former and provided the AuNRs with a good PDT potential. Thus, the phototoxic effect exhibited by the hybrid AuNRs is attributed to the PP-PS component and enhanced by the presence of the AuNRs. Probably, the presence of the PP-PS conjugate onto the water-dispersible AuNRs might have established a better disaggregation of this conjugate. Consequently, this avoided the modification of the photophysical properties of Pyro. Taking into account that the hydrophobicity of Pyro can decrease when linked to the H-KDKPPR-OH peptide of hydrophilic nature, but this should have been further enhanced when the whole conjugate is coupled to AuNRs.

Therefore, the hybrid AuNRs@PEK-MI-K(Pyro)DKPPR-OH nanosystem proves good competences in drug delivery (active and passive) in addition to PDT.

## 4. Conclusions

In summary, AuNRs were successfully synthesized and functionalized with polyethylene glycol (PEG) chains to limit their toxicity. Thiolation of the AuNRs@PEG by Traut’s reagent was then performed. The AuNRs possessed a length of about 44 nm and an average width of about 8 nm. The AuNRs were characterized physicochemically by TEM, Raman spectroscopy, zeta-potential, XPS, etc. H-KDKPPR-OH peptide was also synthesized for the indirect vascular targeting of NRP-1 recombinant protein and further conjugated to phototoxic pyropheorbide-a PS. The final conjugate was coupled to 6-Maleimidohexanoic acid to yield MI-K(Pyro)-DKPPR-OH. The coupling of this conjugate to the surface of the thiolated AuNRs was achieved through the reaction between the maleimide-thiol groups. The photophysical properties of free Pyro, including their absorption profile in the visible range, the good fluorescence intensity and singlet oxygen production was preserved upon coupling to the AuNRs. This nanosystem generated a fluorescence and singlet oxygen quantum yields of 0.4 and 0.3, respectively, upon excitation at 412 nm. They also showed good molecular affinity to NRP-1 recombinant protein (IC_50_ = 1.1 µM), which is comparable to free KDKPPR peptide. In addition, when tested with glioblastoma U87 cells in vitro, they appeared to be biocompatible. However, when treated cells were irradiated at 652 nm, a remarkable photodynamic effect was attained. Under these conditions, 67% of cells were affected upon their exposure to the hybrid AuNRs at 30 µM of Pyro concentration. The efficiency of this hybrid AuNRs in the vascular targeting and PDT was validated. The work described in this publication paves the way for many perspective future continuation and multiple applications of these NRs. On this basis, we can conclude that this nanosystem, with all its components, holds, as a perspective, a potential for combined photodynamic/photothermal therapy due to the presence of Au. Moreover, it can also serve in tumor detection by NIR fluorescence beside the cancer treatment as a theranostic nanosystem.

## Figures and Tables

**Table 1:** Number of polyethylene glycol (PEG) per gold nanorods (AuNRs) obtained from fluorescamine- and ninhydrin-based assays.

	Total PEGs per AuNRs	
HS-PEG-NH_2_ (Dalton)	Measured by the fluorescamine-based assay	Measured by the ninhydrin-based assay
2000	1466 +/− 6	1600 +/− 35

**Table 2:** The photphysical properties of the functionalized AuNRs and the different intermediates.

Compound	λ_max_ (nm)	Φ_f_	Φ_Δ_	τ_f_ (ns)	τ_Δ_ (μs)
Pyro	412	0.38	0.51	6.8	14
Fmoc-K(Pyro)-OH	412	0.35	0.51	6.9	14
H-K(Pyro)DKPPR-OH	412	0.34	0.44	6.8	14
MI-K(Pyro)DKPPR-OH	412	0.29	0.39	6.5	13
AuNRs@PEG-MI-K(Pyro)DKPPR-OH	412	0.30	0.40	6.5	12

## Supplementary Materials

The following are available online at https://www.mdpi.com/2077-0383/8/12/2205/s1. Figure S1: Calibration curves for HS-PEG2K-NH_2_ and fluorescamine-based assay at pH = 10, Figure S2: Calibration curves for HS-PEG2K-NH_2_ and ninhydrin-based assay, Figure S3: HRMS of Pyro-NHS, Figure S4: HRMS of Fmoc-Lys(Pyro)-OH, Figure S5: ^1^H NMR of Fmoc-Lys(Pyro)-OH, Figure S6: COSY and TOCSY spectra of Fmoc-Lys(Pyro)-OH (300 MHz, DMSO-d_6_), Figure S7: HRMS of Fmoc-K(Pyro)DKPPR-OH, Figure S8: ^1^H NMR of Fmoc-K(Pyro)DKPPR-OH, Figure S9: COSY and TOCSY spectra of Fmoc-K(Pyro)DKPPR-OH (300 MHz, DMSO-d_6_), Figure S10: HRMS of MI-K(Pyro)DKPPR-OH, Figure S11: ^1^H NMR (300 MHz, DMSO-d_6_, δ) of MI-K(Pyro)DKPPR-OH, Figure S12: COSY and TOCSY spectra of MI-K(Pyro)DKPPR-OH (300 MHz, DMSO-d_6_), Table S1: Concentrations of HS-PEG2K-NH_2_ taken from the original solution, from the supernatant after incubation and that onto the AuNRs derived by subtraction, Table S2: ^1^H NMR (300 MHz, DMSO-d_6_, δ) of H-DKPPR-OH, Table S3: ^1^H NMR (300 MHz, DMSO-d_6_, δ) of H-K(Pyro)DKPPR-OH, Table S4: ^1^H NMR (300 MHz, DMSO-d_6_, δ) of MI-K(Pyro)DKPPR-OH
