# Supplementary material for: New Targeted Gold Nanorods for the Treatment of Glioblastoma by Photodynamic Therapy
Source: J Clin Med. 2019 Dec 13;8(12):2205. doi: 10.3390/jcm8122205 (PMC6947424; doi:10.3390/jcm8122205)
Supplement: Supplementary file 1 [file jcm-08-02205-s001.pdf]

Article

New Targeted Gold Nanorods for the Treatment of Glioblastoma by Photodynamic Therapy

Zahraa Youssef [1], Yesmurzayeva Nurlykyz [1,2], Ludivine Larue [1], Jouan-Hureaux Valérie [3], Colombeau Ludovic [1], Arnoux Philippe [1], Acherar Samir [4], Régis Vanderesse [4] and Céline Frochot [1,*]

Supporting Materials

Content:

Figure S1. Calibration curves for HS-PEG2K-NH_2 and fluorescamine-based assay at pH = 10. This graph shows a linear relationship between the fluorescence intensity recoded at 480 nm and the concentration of PEG.

Figure S2. Calibration curves for HS-PEG2K-NH_2 and ninhydrin-based assay. This graph shows a linear relationship between the UV-vis absorption recoded at 565 nm and the concentration of PEG.

Figure S3. HRMS of (Pyro)-NHS

Figure S4. HRMS of Fmoc-(Pyro)-Lys-OH

Figure S5. ^{1}H NMR (300 MHz, DMSO-d_6) of Fmoc-(Pyro)-Lys-OH

Figure S6. COSY and TOCSY spectra of Fmoc-(Pyro)-Lys-OH (300 MHz, DMSO-d_6)

Figure S7. HRMS of Fmoc-K(Pyro)DKPPR-OH

Figure S8. ^{1}H NMR (300 MHz, DMSO-d_6) of Fmoc-K(Pyro)DKPPR-OH

Figure S9. COSY and TOCSY spectra of Fmoc-K(Pyro)DKPPR-OH (300 MHz, DMSO-d_6)

Figure S10. HRMS of MI-K(Pyro)DKPPR-OH

Figure S11. ^{1}H NMR (300 MHz, DMSO-d_6, δ) of MI-K(Pyro)DKPPR-OH

Figure S12. COSY and TOCSY spectra of MI-K(Pyro)DKPPR-OH (300 MHz, DMSO-d_6)

Table S1. Concentrations of HS-PEG2K-NH_2 taken from the original solution, from the supernatant after incubation and that onto the AuNRs derived by subtraction.

Table S2. ^{1}H NMR (300 MHz, DMSO-d_6, δ) of H-DKPPR-OH

Table S3. ^{1}H NMR (300 MHz, DMSO-d_6, δ) of H-K(Pyro)DKPPR-OH

Table S4. ^{1}H NMR (300 MHz, DMSO-d_6, δ) of MI-K(Pyro)DKPPR-OH

1. Calibration Curves

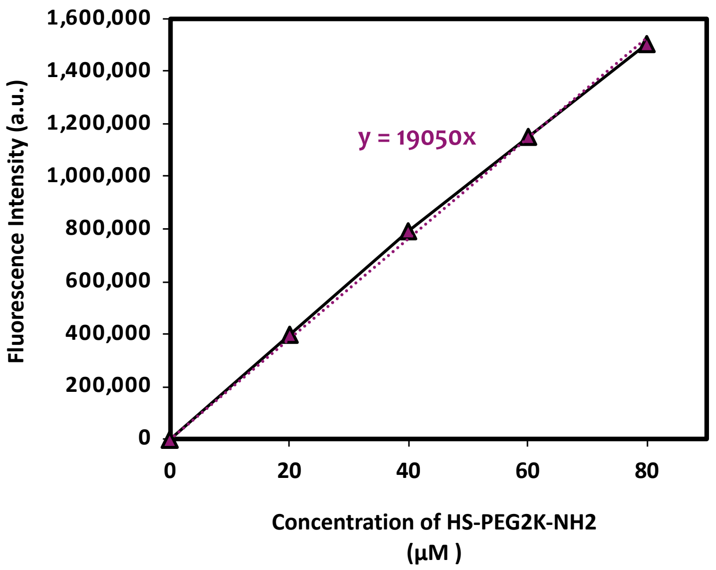

Figure S1. Calibration curves for HS-PEG2K-NH_2 and fluorescamine-based assay at pH = 10. This graph shows a linear relationship between the fluorescence intensity recorded at 480 nm and the concentration of PEG.

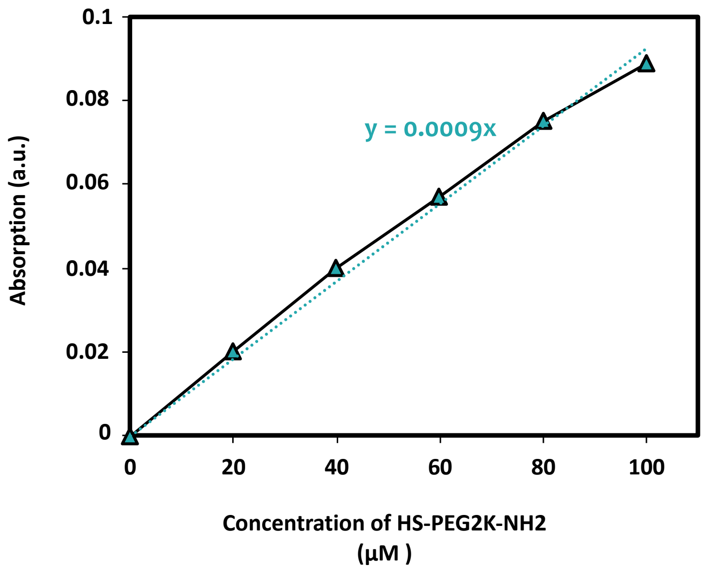

Figure S2. Calibration curves for HS-PEG2K-NH_2 and ninhydrin-based assay. This graph shows a linear relationship between the UV-vis absorption recorded at 565 nm and the concentration of PEG.

Table S1. Concentrations of HS-PEG2K-NH_2 taken from the original solution, from the supernatant after incubation and that onto the AuNRs derived by subtraction.

	HS-PEG-NH_2 (Dalton)	Concentration in the Original Solution (mM)	Concentration Taken from the Supernatant after Incubation (mM)	Concentration of PEG on AuNRs (mM)
Measured by the fluorescamine-based assay	2000	1.6	0.0096	1.587
			0.0038	1.593
Measured by the ninhydrin-based assay	2000	1.6	0.1497	1.446
			0.1094	1.491

2. Characterizations

2.1. (Pyro)-NHS

Data: Rf = 0.70 (CH_2Cl_2/EtOH = 97/3, v/v). **^{1}H NMR** (300 MHz, DMSO-d$_6$, δ): -1.96 and 0.25 (each s, 1H, *NH* pyrrole), 1.61 (t, J = 7.5 Hz, 3H, *CH$_3$*CH$_2$C=), 1.76 (d, J = 7.2 Hz, 3H, *CH$_3$*CH-), 2.70-2.80 (m, 4H, -*CH$_2$CH$_2$*COO-), 2.85 (s, 4H, -*CH$_2$*-), 3.20, 3,42 and 3,59 (each s, 3H, *CH$_3$*C=), 3.68 (m, 2H, CH$_3$*CH$_2$*C=), 4.39 (d, J = 12 Hz, 1H, *CH*CH$_2$CH$_2$COO), 4.68 (m, 1H, CH$_3$*CH*-), 5.08 and 5.21 (each d, J = 20.1 Hz, 1H, =*CH*-CH$_2$CO), 6.20 (d, J = 11.7 Hz, 1H, H$_2$C=*CH*-, cis), 6.37 (d, J = 17.7 Hz, 1H, H$_2$C=*CH*-, trans), 8.2 (dd, J = 18 Hz and J = 11.7 Hz, 1H, H$_2$C=*CH*-), 8.87, 9.42 and 9.70 (each s, 1H, β-H, -C-*CH*=C-). **HRMS** (ESI): m/z calcd. for $C_{37}H_{37}N_5O_5$ [M + H]$^+$ 632.2867; found [M + H]$^+$ 632.2822. **UV/Vis** (EtOH): λ_{max}(log ε) = 410 (4.61), 510 (3.63), 539 (3.61), 607 (3.57), 666 nm (4.28).

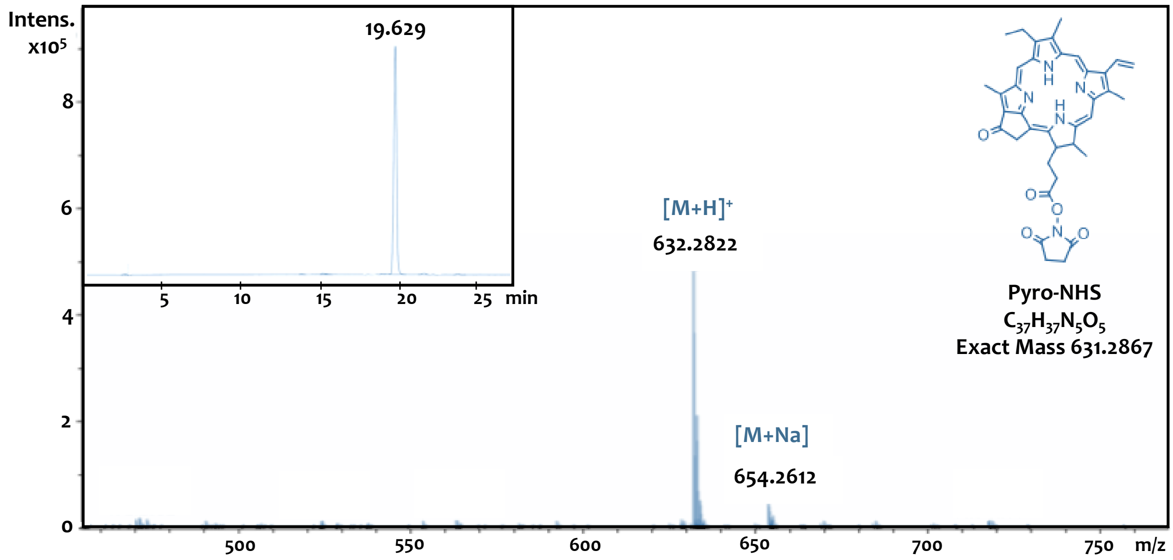

Figure S3. HRMS of (Pyro)-NHS

2.2. Fmoc-(Pyro)-Lys-OH

Data: Rf = 0.40 (CH_2Cl_2/EtOH = 90/10, v/v). **^{1}H NMR** (300 MHz, DMSO-d$_6$, δ): -2.01 and 0.19 (each s, 1H, N*H* pyrrole), 1.27 (m, 2H, γ-*CH$_2$* Lys), 1.54 (m, 4H, β and δ-*CH$_2$* Lys), 1.61 (t, *J* = 7.2 Hz, 3H, *CH$_3$*CH$_2$C=), 1.77 (d, *J* = 7.2 Hz, 3H, *CH$_3$*CH-), 2.09 (m, 2H, -CH*CH$_2$*CH$_2$COO-), 2.30 and 2.58 (each m, 1H, -CHCH$_2$*CH$_2$*COO-), 2.97 (m, 2H, ε-CH$_2$ Lys), 3.19, 3.42 and 3.58 (each s, 3H, *CH$_3$*C=), 3.67 (q, *J* =

7.2 Hz, 2H, $CH_3\underline{CH_2}C$=), 3.79 (m, 2H, α-<u>CH</u> Lys), 3.99 (m, 1H, <u>CH</u> Fmoc), 4.07 (m, 2H, $\underline{CH_2}$ Fmoc), 4.25 (d, J = 8.1 Hz, 1H, $\underline{CH}CH_2CH_2COO$), 4.53 (q, J = 6.9 Hz, 1H, $CH_3\underline{CH}$-), 5.07 and 5.21 (each d, J = 20.1 Hz, 1H, =C-$\underline{CH_2}$CO), 6.18 (d, J = 11.4 Hz, 1H, $\underline{H_2C}$=CH-, *cis*), 6.37 (d, J = 18.0 Hz, 1H, $\underline{H_2C}$=CH-, *trans*), 7.20 and 7.60 (m, 8H, <u>CH</u> phenyl-Fmoc), 7.30 (m, 1H, ε-N*H* Lys), 7.77 (t, 1H, <u>NH</u> Lys), 8.18 (dd, J = 17.7 Hz and J = 11.4 Hz, 1H, H_2C=<u>CH</u>-), 8.84, 9.40 and 9.67 (each s, 1H, β-H, -C-<u>CH</u>=C-), 12.52 (s, 1H, -COO<u>H</u>). **HRMS** (ESI): *m/z* calcd. for $C_{54}H_{56}N_6O_6$ [M + H]$^+$ 885.4334; found [M + H]$^+$ 885.4269. **UV/Vis** (EtOH): λ_{max}(log ε) = 413 (4.92), 512 (3.92), 543 (3.86), 612 (3.87), 669 nm (4.59).

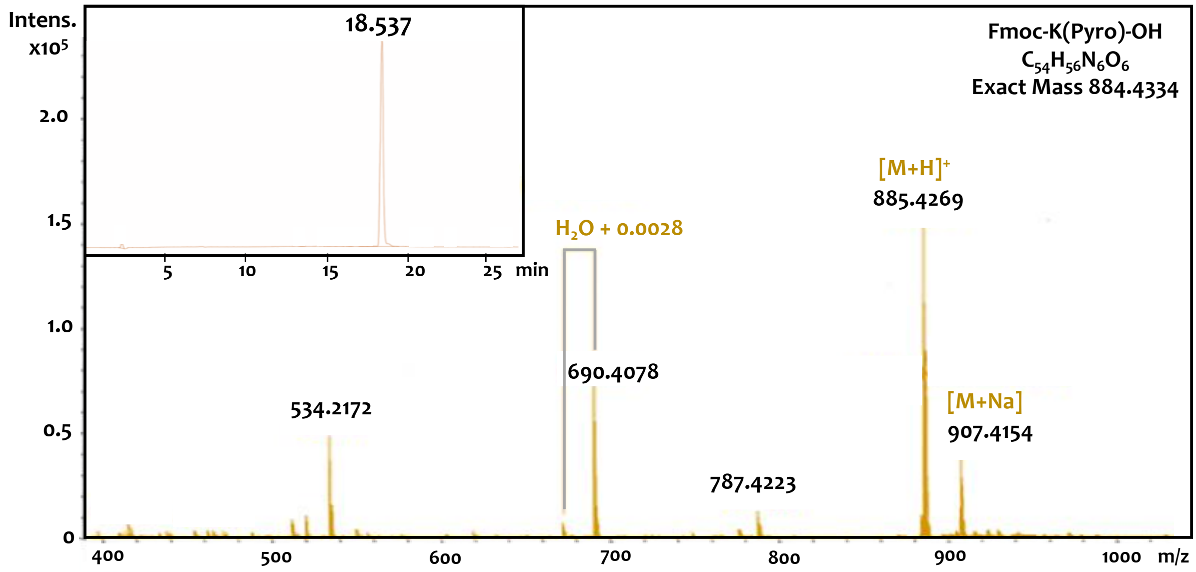

Figure S4. HRMS of Fmoc-(Pyro)-Lys-OH

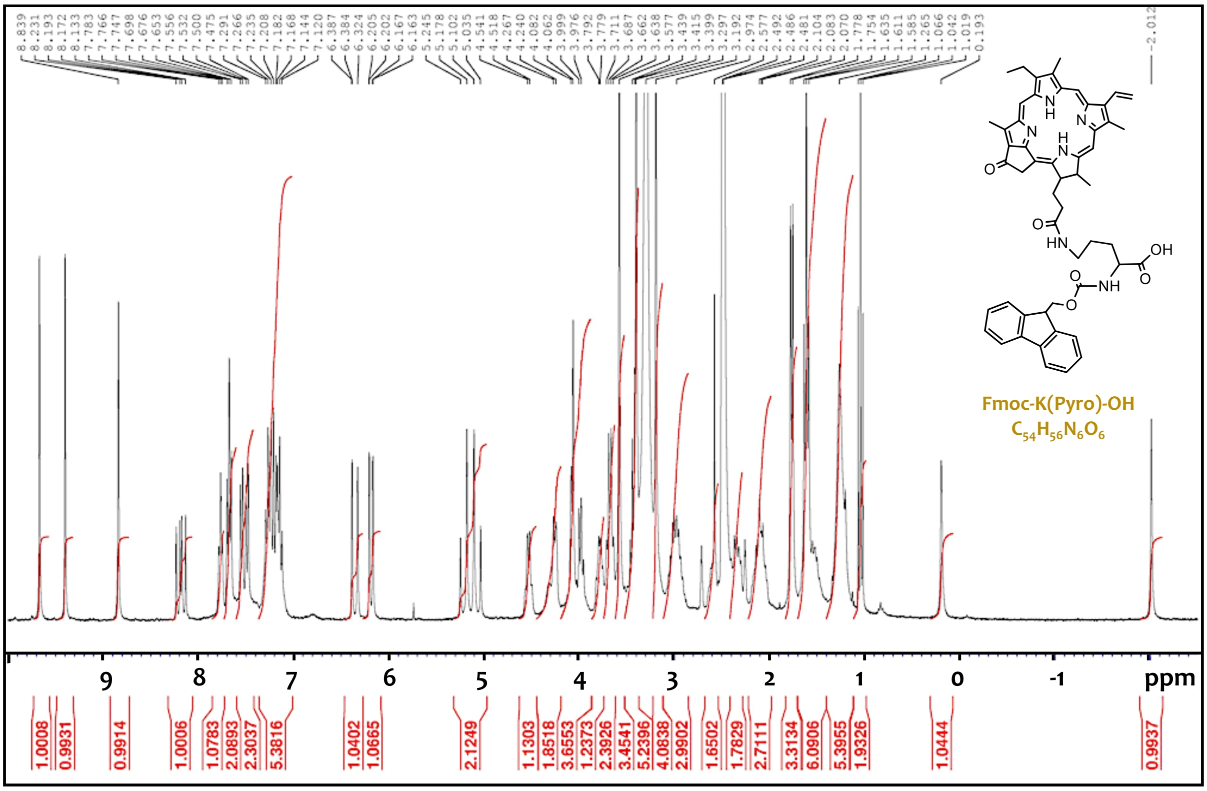

Figure S5. ^{1}H NMR (300 MHz, DMSO-d$_6$) of Fmoc-(Pyro)-Lys-OH

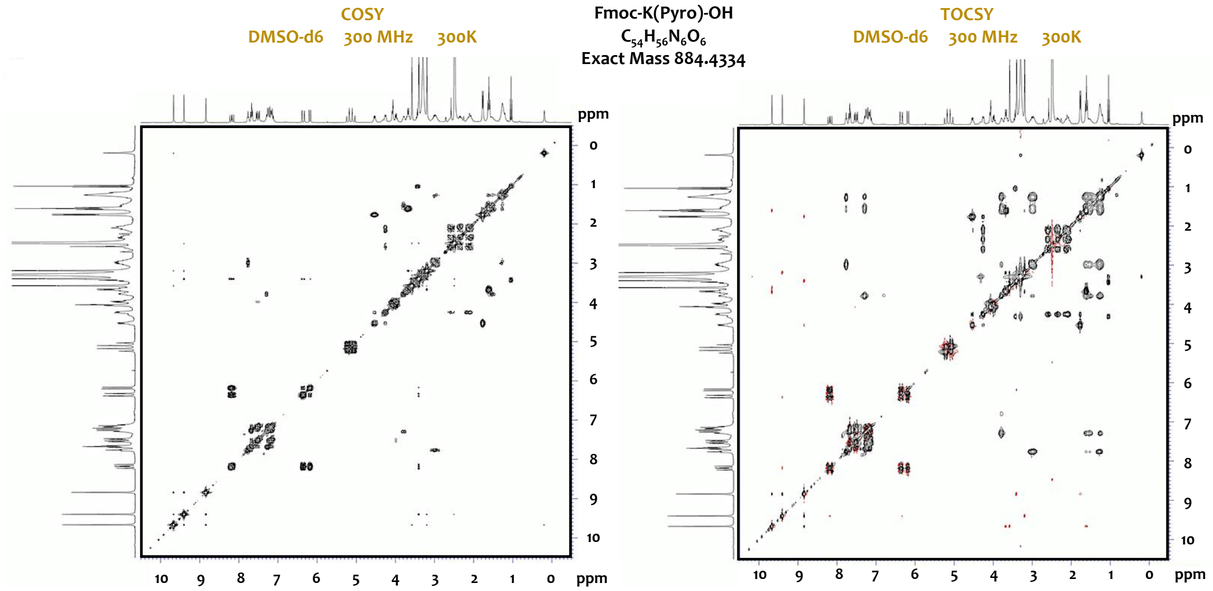

Figure S6. COSY and TOCSY spectra of Fmoc-(Pyro)-Lys-OH (300 MHz, DMSO-d_6)

2.3. H-DKPPR-OH

Table S2. ^{1}H NMR (300 MHz, DMSO-d_6, δ) of H-DKPPR-OH

	NH	α-H	β-H	γ-H	δ-H	Others
Asp		4.12	2.54, 2.58			
Lys	8.23	4.12	1.70	1.25	1.60	ε-NH_3^+ = 8.00; ε-CH_2 = 3.09
Pro		4.55	1.70, 1.90	1.84	3.55, 3.65	
Pro		4.48	1.68, 1.89	1.90	3.52, 3.55	
Arg	8.76	4.44	1.51, 1.60	1.52	3.22	ε-NH = 8.58

2.4. Fmoc-K(Pyro)DKPPR-OH

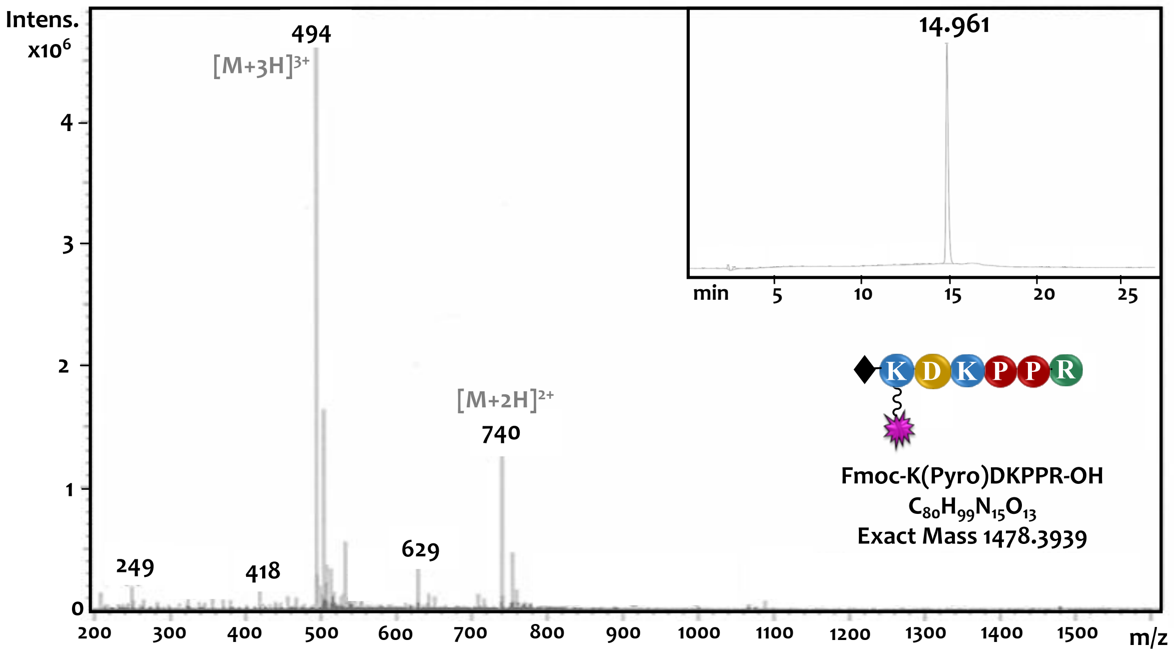

Figure S7. HRMS of Fmoc-K(Pyro)DKPPR-OH

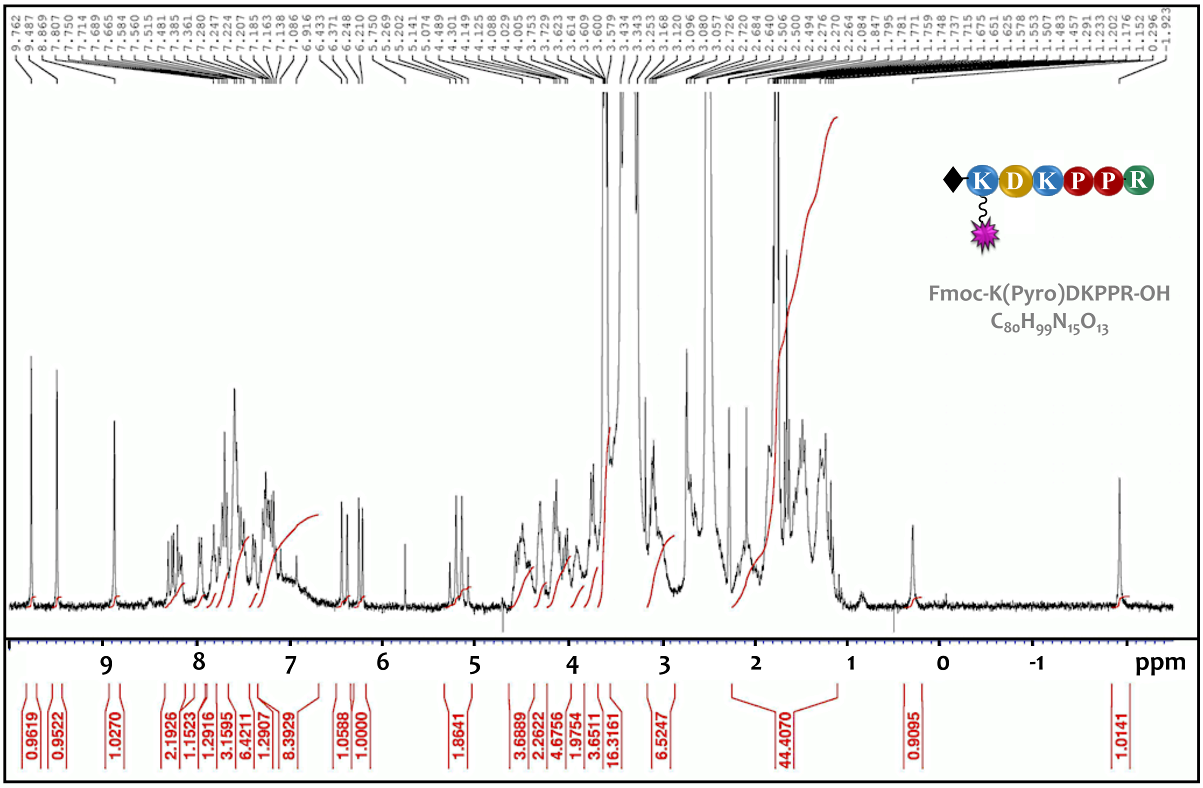

Figure S8. ^{1}H NMR (300 MHz, DMSO-d_6) of Fmoc-K(Pyro)DKPPR-OH

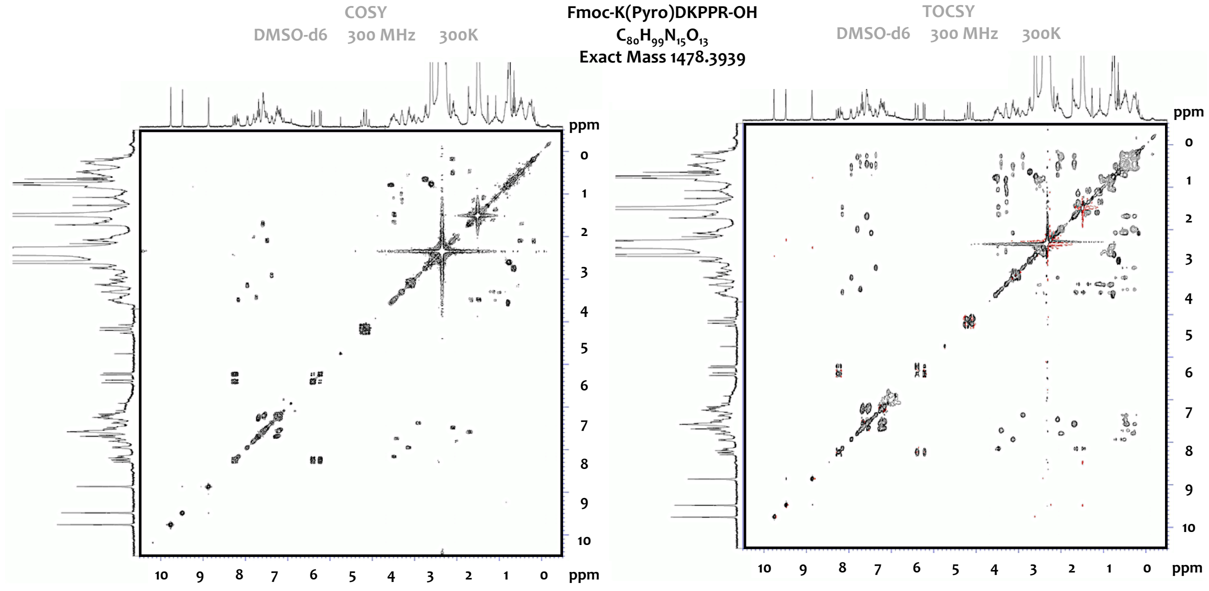

Figure S9. COSY and TOCSY spectra of Fmoc-K(Pyro)DKPPR-OH (300 MHz, DMSO-d_6)

2.5. H-K(Pyro)DKPPR-OH

Data: HRMS (ESI): *m*/*z* calcd. for $C_{65}H_{89}N_{15}O_{11}$ $[M + H]^{+}$ 1256.6939, $[M + 2H]^{2+}$ 628.8506 ; found $[M + H]^{+}$ 1256.6986, $[M + 2H]^{2+}$ 628.8562. **UV/Vis** (EtOH): $\lambda_{max}(\log \varepsilon)$ = 410 (4.54), 510 (3.54), 539 (3.51), 609 (3.48), 667 nm (4.21).

Table S3. ^{1}H NMR (300 MHz, DMSO-d_6, δ) of H-K(Pyro)DKPPR-OH

	NH	α-H	β-H	γ-H	δ-H	Others
Pyro	-	-	-	-	-	-1.93 and 0.29 (s, 2H, NH pyrrole), 1.64 (m, 3H, $\underline{CH_3}CH_2C=$), 1.80 (m, 3H, $\underline{CH_3}CH$-), 2.05 (m, 2H, -$\underline{CH_2}CH_2COO$-), 2.39 and 2.62 (m, 2H, -$\underline{CH_2CH_2}COO$-), 3.23, 3.45 and 3.63 (each s, 3H, $\underline{CH_3}C=$), 3.76 (m, 2H, $CH_3\underline{CH_2}C=$), 4.31 (m, 1H, -$\underline{CH}CH_2CH_2COO$), 4.60 (m, 1H, $CH_3\underline{CH}$-), 5.12 and 5.24 (each d, J = 20.1 Hz, 1H, =C-$\underline{CH_2}CO$), 6.21 (d, J = 11.7 Hz, 1H, $\underline{H_2C}$=CH-, cis) and 6.42 (d, J = 18.3 Hz, 1H, $\underline{H_2C}$=CH-, trans), 8.23 (m, 1H, $H_2C=\underline{CH}$-), 8.90, 9.46 and 9.74 (each s, 1H, β-H, -C-$\underline{CH}$=C-)
Lys1	8.08	4.41	1.63	1.27	1.34	ε-CH_2= 3.03
Asp	8.66	4.55	2.50, 2.68	-	-	
Lys	7.75	4.41	1.58	1.31	1.45	ε-CH_2= 2.72
Pro	-	4.31	1.97, 2.16	1.86	3.45, 3.52	
Pro	-	4.48	1.77, 2.03	1.83	3.41, 3.60	
Arg	7.98	4.13	1.60, 1.73	1.51	3.09	ε-NH= 7.69

2.6. MI-K(Pyro)DKPPR-OH

Data: HRMS (ESI): *m/z* calcd. for $C_{75}H_{101}N_{16}O_{14}$ $[M + H]^+$ 1449.76; found $[M + H]^+$ 1449.7569, $[M + 2H]^{2+}$ 725.3914. **UV/Vis** (EtOH): λ_{max}(log ε) = 412 (4.80), 508 (3.74), 539 (3.70), 610 (3.70), 667 (4.47)

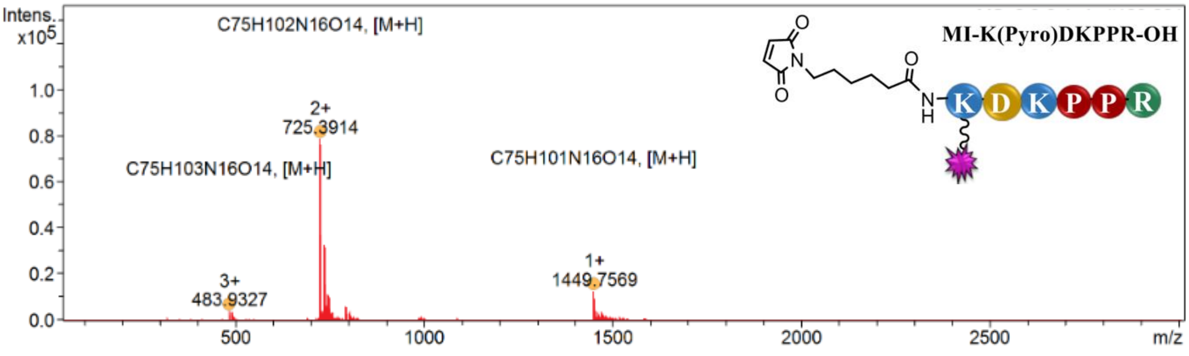

Figure S10. HRMS of MI-K(Pyro)DKPPR-OH

Table S4. ^{1}H NMR (300 MHz, DMSO-d_6, δ) of MI-K(Pyro)DKPPR-OH

	NH	α-H	β-H	γ-H	δ-H	Others
MI	-	-	-	-	-	1.02 (m, 2H, -NCH2CH2*CH2*CH2CH2CONH-), 1.34 (m, 4H, -NCH2*CH2*CH2- and -CH2*CH2*CH2CONH-), 2.00 (m, 2H, -CH2*CH2*CONH-), 3.21 (m, 2H, -N*CH2*CH2-), 6.90 (s, 2H, OC-*CH*=*CH*-CO)
Pyro	-	-	-	-	-	-2.00 and 0.20 (s, 2H, NH pyrrole), 1.59 (m, 3H, *CH3*CH2C=), 1.81 (m, 3H, *CH3*CH-), 2.13 (m, 2H, -*CH2*CH2COO-), 2.39 and 2.63 (m, 2H, -*CH2CH2*COO-), 3.18, 3.44 and 3.60 (each s, 3H, *CH3*C=), 3.68 (m, 2H, CH3*CH2*C=), 4.29 (m, 1H, -*CH*CH2CH2COO), 4.60 (m, 1H, CH3*CH*-), 5.10 and 5.24 (each d, *J* = 20.1 Hz, 1H, =C-*CH2*CO), 6.20 (d, *J* = 11.4 Hz, 1H, *H2C*=CH-, *cis*) et 6.37 (d, *J* = 18.0 Hz, 1H, *H2C*=CH-, *trans*), 8.20 (m, 1H, H2C=C*H*-), 8.87, 9.38 and 9.64 (each s, 1H, β-H, -C-*CH*=C-)
Lys1	7.86	4.10	1.74	1.22	1.52	ε-CH2= 2.99
Asp	8.10	4.48	2.50, 2.66	-	-	
Lys	7.68	4.42	1.71	1.30,	1.52	ε-CH2= 2.73
Pro	-	4.34	1.84, 2.01	1.91	3.49, 3.57	
Pro	-	4.51	1.78, 2.08	1.85	3.43, 3.61	
Arg	7.98	4.15	1.61, 1.74	1.50	3.11	ε-NH= 7.70

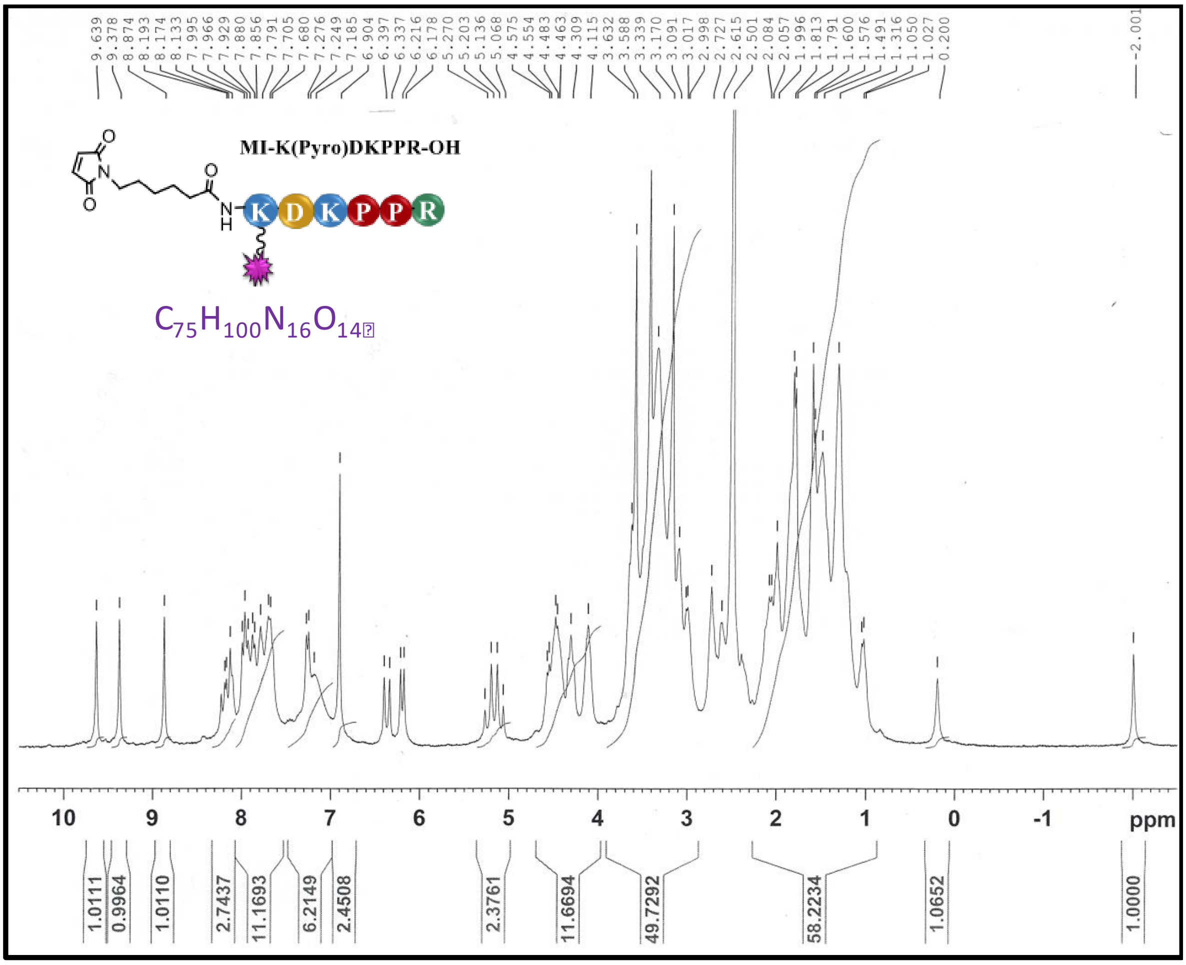

Figure S11. **¹H NMR** (300 MHz, DMSO-d₆, δ) of MI-K(Pyro)DKPPR-OH

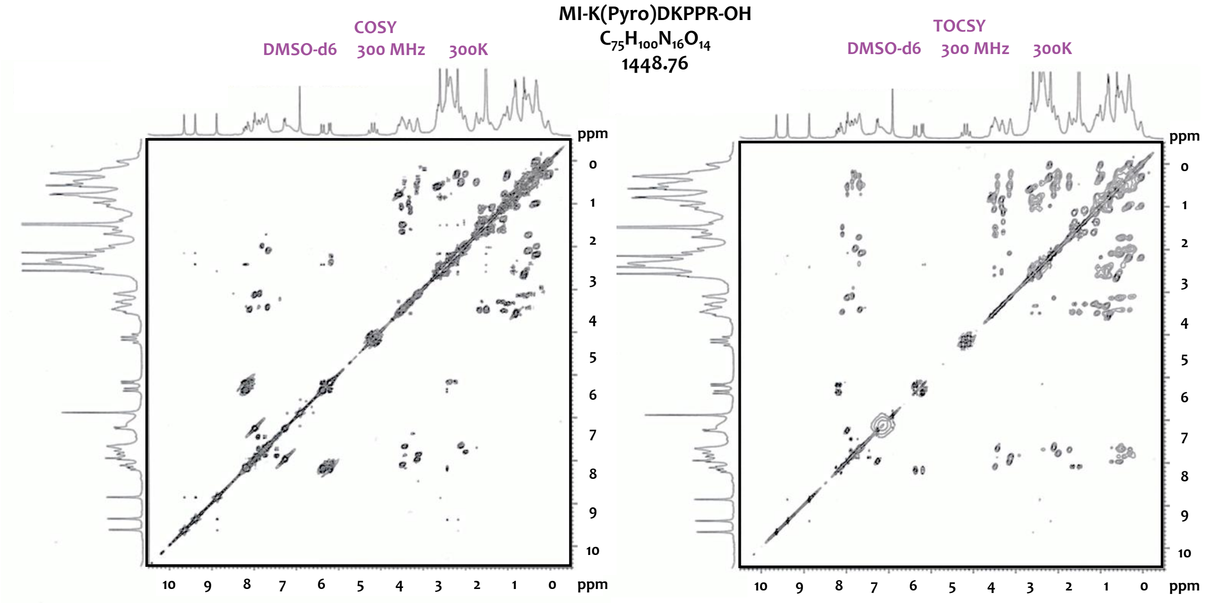

Figure S12. COSY and TOCSY spectra of MI-K(Pyro)DKPPR-OH (300 MHz, DMSO-d₆)
